# Neural Mechanisms Underlying Repetitive Behaviors in Rodent Models of Autism Spectrum Disorders

**DOI:** 10.3389/fncel.2020.592710

**Published:** 2021-01-14

**Authors:** Tanya Gandhi, Charles C. Lee

**Affiliations:** Department of Comparative Biomedical Sciences, Louisiana State University School of Veterinary Medicine, Baton Rouge, LA, United States

**Keywords:** autism models, repetitive behavior, neural mechanisms, signaling, circuitry, neuroanatomical alterations

## Abstract

Autism spectrum disorder (ASD) is comprised of several conditions characterized by alterations in social interaction, communication, and repetitive behaviors. Genetic and environmental factors contribute to the heterogeneous development of ASD behaviors. Several rodent models display ASD-like phenotypes, including repetitive behaviors. In this review article, we discuss the potential neural mechanisms involved in repetitive behaviors in rodent models of ASD and related neuropsychiatric disorders. We review signaling pathways, neural circuits, and anatomical alterations in rodent models that display robust stereotypic behaviors. Understanding the mechanisms and circuit alterations underlying repetitive behaviors in rodent models of ASD will inform translational research and provide useful insight into therapeutic strategies for the treatment of repetitive behaviors in ASD and other neuropsychiatric disorders.

## Introduction

Autism spectrum disorder (ASD) consists of a group of neurodevelopmental disorders with shared, yet heterogeneous, behaviors. With the introduction of improved diagnostic criteria, there has been a substantial rise in the prevalence of autistic cases in the last few decades, reported between three and six children per 1,000 worldwide (Kassim and Mohamed, 2019; Lord et al., 2020) and 1 in 54 children in the US (Zablotsky et al., 2019; Maenner et al., 2020). The variability in global prevalence is largely due to differences in methodological assessment and environmental and/or geographical factors (Chiarotti and Venerosi, 2020; Lord et al., 2020). Both genetic and environmental factors influence the development of ASD and may converge on similar neural outcomes, such as altered connectivity, excitation/inhibition imbalance, and signaling system alterations (Muhle et al., 2004; Satterstrom et al., 2020). Several candidate genes have been associated with the development of ASD (Levitt and Campbell, 2009; Yuen et al., 2017; Feliciano et al., 2019; Grove et al., 2019; Guo et al., 2019); siblings born in families with ASD are particularly high risk indicating a strong genetic basis (Stubbs et al., 2016). Environmental factors involved in the development of ASD include prenatal and postnatal complications, viral infections and nutrient deficiencies (Grabrucker, 2013; Sealey et al., 2016; Karimi et al., 2017; Modabbernia et al., 2017). Understanding these environmental and genetic interactions in autism risk will help guide treatment strategies for ASD (Chaste and Leboyer, 2012; LaSalle, 2013; Tordjman et al., 2014; Kim and Leventhal, 2015; Nardone and Elliott, 2016).

Children with ASD are characterized by social and communication challenges and restricted, repetitive behaviors (Baranek, 1999; Lord et al., 2000). These core behaviors are often accompanied by comorbidities such as epilepsy, anxiety, hyperactivity, and aggression (Richler et al., 2007; King et al., 2009). The restricted, repetitive behaviors (RRBs) in ASD are clustered into two categories. The repetitive behaviors include stereotypic motor movements, repetitive use of objects, self-injurious behaviors, and the circumscribed behaviors include compulsions, desire for sameness, rituals, and restricted interests (Zandt et al., 2007; Whitehouse and Lewis, 2015). The restricted, repetitive behaviors in ASD share similarities with obsessive-compulsive disorder (OCD) and other neuropsychiatric and neurodevelopmental disorders (Scahill and Challa, 2016; Jiujias et al., 2017; Gulisano et al., 2020). Currently, behavioral and pharmacological interventions target specific symptoms and/or associated comorbidities, which are personalized according to individual needs (Eissa et al., 2018; Chahin et al., 2020). Yet, more robust therapeutic interventions have been required that target the underlying neural mechanisms that govern these core autistic symptoms.

Behavioral approaches are typically used to treat repetitive behaviors in ASD and related neurodevelopmental disorders. Behavioral approaches usually employ reinforcement procedures, altering the environment, and promoting variability and flexibility in behavior (Boyd et al., 2012). Pharmacological interventions for irritability and some forms of repetitive behavior, such as self-injurious behavior include selective serotonin reuptake inhibitors (SSRIs) like Fluoxetine and antipsychotics such as haloperidol (typical) and Risperidone (atypical) (Gencer et al., 2008; Miral et al., 2008; Malone and Waheed, 2009; Doyle and McDougle, 2012; DeFilippis and Wagner, 2016; Masi et al., 2017; Maneeton et al., 2018). Risperidone is a second-generation antipsychotic medication that has been FDA approved for the treatment of irritability in children and adolescents (McDougle et al., 2005, 2008; Scahill et al., 2007, 2012; Aman et al., 2009). It is an antagonist at the serotonin 2A and dopamine D2 receptors and is useful in alleviating irritability, aggression, and self-injurious behavior in young ASD subjects (McCracken et al., 2002; Shea et al., 2004; Chavez et al., 2006; Kent et al., 2013; Fung et al., 2016; Maneeton et al., 2018). Besides, in controlled clinical trials, some of these pharmacological medications also reduce repetitive behaviors, but with potential side-effects that limit the widespread usage of these drugs in the treatment of ASD and as such is not approved by the FDA for repetitive disorders (McPheeters et al., 2011; Sharma and Shaw, 2012; Whitehouse and Lewis, 2015). Additionally, the benefits of pharmacological medications in improving ASD behavior are highly variable across studies and clinical populations. There is also a paucity of long-term clinical trials with a large sample size on pharmacological interventions against restricted/repetitive behavior in ASD (Yu et al., 2020; Zhou et al., 2020). Furthermore, there is a lack of evidence-based treatment strategies targeting diverse repetitive/restricted behaviors in ASD. Hence, novel treatment strategies are required that target core autistic deficits, while limiting the detrimental side effects of such medications. In this review article, we have discussed preclinical studies demonstrating the efficacy of the pharmacological treatments on restricted/repetitive behaviors, which are still under development for targeting repetitive/restricted behaviors in a clinical population. Besides, we have also reviewed studies pointing in the direction of circuit-based strategies for targeting repetitive/restricted behaviors in rodent models of ASD.

As an approach to developing new therapeutics, several rodent models of ASD have been generated with good construct validity that recapitulates many of the behavioral phenotypes observed in autistic individuals. The behavioral tasks assessing repetitive behaviors are more developed than behavioral tasks assaying resistance to change or restricted behaviors (Lewis et al., 2007). The studies we will review mainly discuss rodent models primarily displaying lower-order stereotyped motor behaviors, which are generally better characterized and easier to model than models of insistence on sameness or restricted behaviors (higher-order). Nevertheless, in this review article, we have also discussed a few rodent models that show both the repetitive and restricted behavioral phenotypes. The repetitive behaviors observed in rodent models of autism are complex and diverse, including self-grooming, jumping, circling, marble burying, hanging, rearing, and forelimb movements and involve several molecular and neural pathways (Whitehouse and Lewis, 2015; Kim et al., 2016). Also, complex restricted behaviors such as resistance to change and narrow interests represent cognitive rigidity to routines and obsessions that correspond with executive function deficits (Lopez et al., 2005). Behavioral assays for resistance to change or cognitive inflexibility in rodents include response extinction, reversal learning, and set-shifting tasks, assessing the inability to change the developed spatial habit (Colacicco et al., 2002; Roullet and Crawley, 2011). Understanding of the complex neural mechanisms underlying repetitive behaviors in these models is expected to boost translational research and provide valuable insight into potential treatments for repetitive behaviors observed in ASD. Therefore, in this review article, we will discuss the underlying mechanisms that mediate the complex motor activities and consequent repetitive behavioral repertoire in different rodent models of ASD.

## Rodent Models of Autism: Genetic Mutations, Environmental Risk Factors, and Some Inbred Strains Displaying Repetitive/Restricted Behaviors

**Figure 1:**
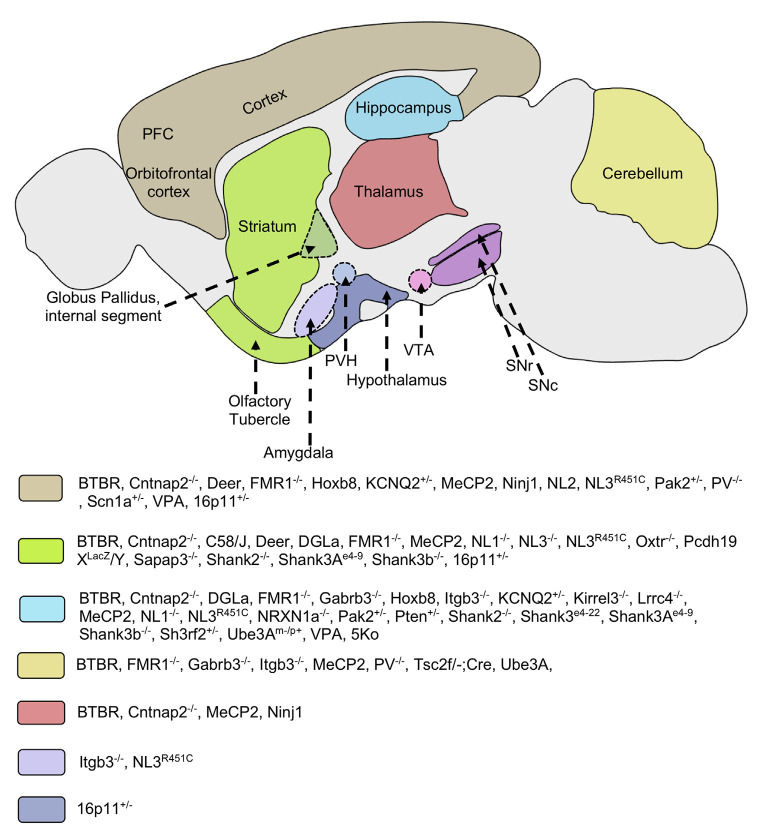
Implicated brain regions in mouse models of autism. Different mouse models of autism exhibit alterations in various brain areas such as the striatum, cortex, thalamus, hippocampus, cerebellum, hypothalamus, and amygdala. These brain regions are involved in cortico-striatal and limbic circuitry. Molecular and/or neuroanatomical changes in these structures are correlated with the pathophysiology of repetitive behaviors. Some mice models implicate multiple brain regions in the pathology of restricted/repetitive behaviors. PFC, prefrontal cortex; VTA, ventral tegmental area; SNc, substantia nigra pars compacta; SNr, substantia nigra pars reticulata; PVH, paraventricular nucleus of hypothalamus; Cntnap2, Contactin Associated Protein-like 2 gene; FMR1, Fragile X mental retardation 1; Gabrb3, Gamma-aminobutyric acid receptor subunit beta-3; Hoxb8, Homeobox protein; Itgb3, Integrin beta-3; KCNQ, Potassium voltage-gated channel subfamily; Kirrel3, Kin of Irregular Chiasm-like 3; Lrrc4, Leucine-rich repeat-containing 4; MeCP2, Methyl CpG binding protein 2; Ninj1, Nerve injury-induced protein-1; NL, Neuroligin; NRXN1a, Neurexin 1a; Oxtr, Oxytocin receptor; Pcdh19, Protocadherin-19; PV, Parvalbumin; Pak2, p21 activated kinase 2; Pten, Phosphatase and tensin homolog; Sapap3, Synapse-associated protein 90/postsynaptic density protein 95 associated protein 3; Shank, SH3 and multiple ankyrin repeat domains 3; Sh3rf2, SH3 Domain Containing Ring Finger 2; Scn1, Sodium Voltage-Gated Channel Alpha Subunit 1; Tsc2, Tuberous Sclerosis Complex 2; Ube3A, Ubiquitin Protein Ligase E3A; VPA, Valproic acid; 5Ko, 5 kainate receptor subunit.

Genetic mutations account for a significant proportion of ASD risk (Ronemus et al., 2014). Genetic mutations in ASD are complex and diverse depending on structure type [i.e., large-scale chromosome abnormalities, small scale insertions, deletions, substitutions, copy number variation (CNV) and single nucleotide variation (SNV)], inheritance type [i.e., germline, somatic, *de novo* mutation (non-inherited)], frequencies (i.e., common, rare and very rare) and protein sequence affected (i.e., frameshift mutation, point substitution (De Rubeis and Buxbaum, 2015; De La Torre-Ubieta et al., 2016; Ramaswami and Geschwind, 2018). Over the last decade, with the advancement of sequencing technology, many genes have been implicated in autism pathogenesis (Geschwind and State, 2015). This review covers many of the most common of these factors, which underscores the range of molecular and cellular factors implicated in ASD. Such diversity of neurobiological factors in ASD further highlights the challenges of treatment development, where seemingly divergent neural factors may converge on similar behavioral outcomes, i.e., restrictive and repetitive behaviors. When possible, we have attempted to highlight some of these similarities and differences in risk factors (Figure 1), which remains a major challenge for the field to define and address.

Many genes are linked to syndromic ASD, in which monogenic syndromes exhibit phenotypic overlap with ASDs (i.e., ASD is secondary to a known genetic cause and disorder with clinically defined presentation) (Walsh et al., 2008; Schaefer and Mendelsohn, 2013; Ramaswami and Geschwind, 2018). Monogenic disorders accounted for in ASD include Fragile X Syndrome (FMR1), Tuberous Sclerosis (TSC1, TSC2), Angelman and Prader-Willi Syndromes (15q11–q13 deletion/UBE3A and GABRB3 deletion), Rett Syndrome (MECP2), Phelan-McDermid syndrome (PMS; 22q13.3 deletion/SHANK3 mutation), Smith-Lemli-Opitz Syndrome (DHCR7), Neurofibromatosis (NF1), Timothy Syndrome (CACNA1C), et cetera (Muhle et al., 2004; Moss and Howlin, 2009; Geschwind, 2011; Ramaswami and Geschwind, 2018). Whereas in idiopathic autism, the cause is unknown.

Susceptibility genes linked with non-syndromic autism involve multiple common and rare variants (CNVs), and *de novo* mutations. This genetic heterogeneity is associated with idiopathic ASD and accounts for a substantial fraction of autism risk, indicating the involvement of multiple genetic pathways in its etiology (Swanwick et al., 2011; Devlin and Scherer, 2012). Multiple genes with different functions implicated in ASD include SHANK1, 2, CNTNAP2, NLGN, NRXN, 16p11.2 microdeletion/microduplication, SCN1A, et cetera (Cook and Scherer, 2008; Geschwind and State, 2015; Ramaswami and Geschwind, 2018; Sultana et al., 2018). Most ASD related genes affect neural circuit structure and function, with defects in either a single neural circuit component (localized) or multiple neural systems (distributed) impacting overall network activity (Figure 1) (Rubenstein, 2010). These neurodevelopmental defects can lead to abnormal neural structure and connectivity, as well as alterations to neurotransmitter systems and their receptors.

Animal models of repetitive and restricted behaviors are classified into different categories by causal factors. The categories of models of repetitive and restricted behavior include: (1) after CNS insult (e.g., specific genetic mutations, lesions or environmental factors); (2) caused by pharmacological agents [e.g., apomorphine (dopamine agonist), amphetamine, cocaine, NMDA (glutamate receptor ligand)]; (3) resulting from restricted housing (e.g., laboratory cage, social deprivation); and (4) linked with particular inbred rodent strains (BTBR, C58) (Lewis et al., 2007; Bechard and Lewis, 2012).

Many of the genetic and environmental factors implicated in the etiology of autism have been modeled using rodents. However, not all rodent models of ASD manifest repetitive behavior. For example, mice with knockout of neuroligin-2 and -4 genes or mutations of the Scn2a (Scn2a^+/–^) gene do not exhibit alterations in intensity or frequency of repetitive behavior (El-Kordi et al., 2013; Wöhr et al., 2013; Shin et al., 2019; Cao et al., 2020). Hence, we will review preclinical studies with particular emphasis on rodent models displaying robust stereotypic behavior (Table 1), as discussed below.

**Table 1:** Neural alterations underlying repetitive behaviors and rescue of repetitive behaviors in rodent models of autism spectrum disorders (ASDs).

Model	Repetitive and related behaviors	Neural alterations	Rescue of repetitive behaviors	References
*BTBR T+tf/J*	• Repetitive self-grooming • Increased marble-burying behavior • Reversal learning deficit in Morris water maze (MWM)	• Reduced GABAergic inhibitory transmission • Upregulation of serotonin 5HT_2A_ receptor density and activity • Increased in glutamatergic transmission in cortico-striatal circuitry • Impaired dopamine D2 receptor function • Reduced expression of BDNF in hippocampus and cortex • Absence of corpus callosum, lack of hippocampal commissure • Reduced cortical thickness • Reduced cerebral white and gray matter • Impaired cortico-thalamic function • Altered volumes of cerebellum, brainstem, striatum, and hippocampus	• mGluR5 receptor antagonist (MPEP) • Selective GABA_b_ receptor agonist (R-baclofen) • Dorsomedial striatal injection of selective 5HT_2A_ receptor antagonist (M100907) • Risperidone • Muscarinic receptor (mAChR) agonist (Oxotremorine) • Nicotinic receptor (nAChR) agonist (nicotine) • Acetylcholinesterase inhibitor (AChEI; Donepezil) reduced behavioral rigidity in water T-maze task • Retinoic acid receptor-related orphan receptor alpha (ROR a) agonist (SR1078)	Wahlsten et al. (2003), Moy et al. (2007), McFarlane et al. (2008), Silverman et al. (2010, 2012), Gould et al. (2011), Wöhr et al. (2011), Amodeo et al. (2012), Burket et al. (2013), Dodero et al. (2013); Ellegood et al. (2013), Reynolds et al. (2013), Han et al. (2014), Karvat and Kimchi (2014), Wang et al. (2015), Wang Y. et al. (2016), and Meyza and Blanchard (2017)
*Cntnap*2^−/−^	• Repetitive self-grooming and digging • Reversal learning deficit (MWM) • Hyperactivity • Seizures	• A decrease in parvalbumin-positive interneurons in striatum resulting in altered activity of the cortico-striatal-thalamic pathway • Cortical migration abnormalities	• Dopamine D2 receptor antagonist (Risperidone)	Peñagarikano et al. (2011) and Lauber et al. (2018)
*C58/J*	• Repetitive self-grooming • Hind limb jumping • Backflips • Decreased exploratory behavior • Reversal learning deficit	• Increased mGluR5 signaling • NMDA receptor hyperfunction • Reduced GABAergic signaling • Reduced dendritic spines • Increased dopaminergic function and cortical activation • Aberrant hippocampal and cortical activity	• mGluR5 negative allosteric modulator (GRN-529) • Selective GABA_b_ receptor agonist (R-baclofen) • Environmental enrichment	Moy et al. (2008b), Ryan et al. (2010), Muehlmann et al. (2012), Silverman et al. (2012), and Whitehouse et al. (2017)
*Deer*	• Repetitive hindlimb jumping and backflips • Perseverative behavior in a reversal-learning task (T-maze)	• Enhanced Cortico-striatal glutamatergic projections • Decrease density of serotonin transporters in the striatum • Reduced indirect basal ganglia pathway activity • Dorsomedial striatum alterations	• Striatal injections of NMDA receptor antagonist (MK-801) • Dopamine D1 receptor antagonist (SCH23390) • Co-administration of adenosine A_2A_ receptor agonist (CGS21680) and A_1_ receptor agonist (CPA) • Selective SSRI (Escitalopram) • Triple drug cocktail (D2R antagonist L-741, 626 + Adenosine A_2A_R agonist CGS21680 + mGluR5 positive allosteric modulator CDPPB) • Environmental enrichment (EE)	Presti et al. (2003); Tanimura Y. et al. (2010), Tanimura et al. (2008, 2011), Wolmarans et al. (2013), Bechard et al. (2017), and Lewis et al. (2019)
*DGLa*^flx/flx^	• Repetitive self-grooming	• Reduced levels of 2-acyl glycerol in the striatum • Excessive glutamatergic drive in direct-pathway MSNs		Shonesy et al. (2014, 2018)
*EphA2/A3 double KO*	• Stereotypic facial grooming • Reduced locomotor activity • Increased pre-pulse inhibition of acoustic startle	• Sensorimotor gating abnormalities • Altered excitability of forebrain pathways		Qiu et al. (2012) and Wurzman et al. (2015)
*FMR1*^−/−^	• Repetitive self-grooming • Increased/decreased marble-burying • A deficit in novelty preference (T-maze spontaneous alternation) • Learning task deficits • Hyperactivity • Anxiety • Reduced motor learning • Olfactory learning deficits	• Increased mGluR-LTD in hippocampal CA1 and cerebellum • Increased endocannabinoid mediated transmission at GABAergic synapses of the hippocampus and dorsal striatum • Dysfunctional cortico-striatal circuitry • Decrease activity of fast-spiking interneurons in cortical areas (hyperexcitability) • Abnormal sensorimotor gating • Altered dendritic spine density and morphology • Impaired long-term potentiation • PSD-95 protein deficits • PI3K/AKT pathway abnormal activity • AMPAR and NMDAR dysfunction • Purinergic signaling alteration • Altered cerebellar and striatal volumes	• Selective GABA-B receptor agonist (R-baclofen) • mGluR5 receptor antagonist (MPEP) • Minocycline (antibiotic inhibiting MMP9) • Antipurinergic therapy (suramin) • CB1R antagonist (rimonabant) • Small-molecule PAK [p21-activated kinase regulates actin cytoskeleton dynamics] inhibitor (FRAX486) • BDNF application • Gene therapy with human FMR1 • Delta-subunit containing extrasynaptic GABA-A receptors agonist (Gaboxadol) • Intracranial injection of CRISPR-Gold targeting mGluR5 • Chronic application of Bryostatin-1 (Protein Kinase C potent activator) • eFT508, MNK (mitogen-activated protein kinase interacting protein kinase) inhibitor • BPN14770, phosphodiesterase-4D negative allosteric modulator (PDE4DNAM) • GSK6A (PI3K antagonist) • FS-115, S6KI (mTORC1-p70 ribosomal S6 kinase 1) inhibitor	Peier et al. (2000), Spencer et al. (2005, 2008), Lauterborn et al. (2007), Dölen and Bear (2008), Errijgers et al. (2008), McNaughton et al. (2008), Paylor et al. (2008), Bilousova et al. (2009), Zhang and Alger (2010), Pietropaolo et al. (2011), Henderson et al. (2012), Jung et al. (2012), Thomas et al. (2012), Busquets-Garcia et al. (2013), Dolan et al. (2013), Berry-Kravis (2014), Gandhi et al. (2014), Naviaux et al. (2015), Tang and Alger (2015), Bhattacharya et al. (2016), Gurney et al. (2017), Sinclair et al. (2017), Lee et al. (2018), Nolan and Lugo (2018), Yau et al. (2018), Zerbi et al. (2018), Cogram et al. (2019, 2020), Gross et al. (2019), and Shukla et al. (2020)
*Gabrb*3^−/−^	• Repetitive circling • Hyperactivity	• Cerebellar vermis hypoplasia • Abnormal GABA-A receptor function in the hippocampus • Altered GABA-A receptor-mediated neurotransmission		DeLorey et al. (1998, 2008), Mercer et al. (2016), and Orefice et al. (2016)
*Hoxb8 KO in microglia*	• Increased grooming • Anxiety-like behavior	• Increased cortical dendritic spine density • Increased dendritic spines in the striatum • Defects in LTP, miniature postsynaptic currents	• Fluoxetine (SSRI)	Greer and Capecchi (2002), Chen et al. (2010), and Nagarajan et al. (2018)
*Itgb3*^−/−^	• Increased grooming in a novel environment	• Alterations in axon/dendrite outgrowth, cell adhesion, and synapse formation • The reduced corpus callosum, hippocampus, striatum, and cerebellum • Increased amygdala volume		De Arcangelis and Georges-Labouesse (2000), Clegg et al. (2003), Carter et al. (2011), and Ellegood et al. (2012)
*KCNQ2*^+/−^	• Repetitive grooming • Hyperactivity • Increased locomotor activity	• Increased neuronal excitability		Yue and Yaari (2006), Shah et al. (2008), Brown and Passmore (2009), and Kim et al. (2020)
*Kirrel3*^−/−^	• Repetitive rearing behavior • Increased locomotor activity • Hypersensitivity to acoustic startle (acoustic startle test) • Hyperactivity	• Abnormal hippocampal mossy fiber synapse formation • Increased CA3 neuron activity during development • Abnormal neuronal migration		Gerke et al. (2006), Serizawa et al. (2006), Nishida et al. (2011), Prince et al. (2013), Martin et al. (2015), Choi et al. (2015), and Hisaoka et al. (2018)
*Lrrc4*^−/−^	• Repetitive self-grooming • Impaired spatial learning (MWM)	• Reduced NMDA receptor-mediated synaptic plasticity • Abnormal synaptic transmission	• NMDA receptor agonist (D-cycloserine)	DeNardo et al. (2012), Soto et al. (2013, 2018), and Um et al. (2018)
*MeCP2*	• Repeated forelimb movements • Deficits in motor coordination and motor learning • Memory deficits	• Decreased levels of dopamine transporter (DAT) and tyrosine hydroxylase (TH) in the striatum • Altered cortical and cerebellar volumes • Cortical LTP deficit • Decreased cortical BDNF levels • Impaired PI3K/AKT/mTOR pathway • Upregulated CB1 and CB2 receptor levels • Hippocampal circuit dysfunction		Shahbazian et al. (2002), Moretti et al. (2005), Lonetti et al. (2010), Lu et al. (2016), Allemang-Grand et al. (2017), and Zamberletti et al. (2019)
*Ninj1*	• Excessive grooming inducing hair loss and lesions • Increased anxiety-like behavior	• Altered synaptic function in thalamocortical neurons • Increased expression of ionotropic glutamate receptor • The increased amplitude of miniature EPSCs	• Fluoxetine (SSRI)	Le et al. (2017)
*NL1*^−/−^	• Repetitive self-grooming • Spatial learning deficits	• Reduced NMDA/AMPA receptor ratio in the hippocampus and dorsal striatum • Reduced hippocampal LTP • Abnormal function of dopamine D1 MSNs • Reduced GluN2A containing NMDARs expression in direct-pathway MSNs • Reduced frequency of miniature excitatory neurotransmission in indirect-pathway MSNs	• NMDA receptor partial co-agonist (D-cycloserine)	Blundell et al. (2010) and Espinosa et al. (2015)
*NL2* overexpression	• Repetitive Jumping	• Reduced E/I balance in PFC		Hines et al. (2008)
*NL3*^−/−^	• Repetitive motor routine • Hyperactivity	• Reduced striatal synaptic function in nucleus accumbens/ventral striatum • Abnormal function of dopamine D1 MSNs • Altered GABAergic signaling and E/I balance in CA2 hippocampal area • Altered synaptic activity in the hippocampus, somatosensory cortex, and basolateral amygdala • Increased AMPA mediated neurotransmission and LTP in the hippocampus		Radyushkin et al. (2009), Rothwell et al. (2014), Modi et al. (2019), Burrows et al. (2015), Hosie et al. (2018), and Matta et al. (2020)
*NL3*^R451C^	• Repetitive behavior (object exploration task) • Aggression	• Smaller striatal volume • Increased striatal postsynaptic density 95 (PSD-95) protein levels	• Risperidone, CB1 receptor agonist (WIN55, 212–2) targeting aggression	Tabuchi et al. (2007), Etherton et al. (2011), and Kumar et al. (2014)
*NRXN1a*^−/−^	• Repetitive self-grooming • Altered nest building • Impaired prepulse inhibition • Aggressive behaviors • Mild anxiety-like behavior	• A decrease in miniature excitatory postsynaptic current frequency in the hippocampus • Impaired excitatory synaptic transmission in the hippocampus • Sensorimotor gating impairments • Increased cortical volume and decreased cerebellar volume		Etherton et al. (2009) and Grayton et al. (2013)
*Oxtr*^−/−^	• Cognitive inflexibility in the reversal phase in T—maze • Increased aggression	• Alterations in excitatory synaptic markers (PSD-95, gephyrin scaffolding proteins) • Altered glutamatergic and GABAergic receptors		Sala et al. (2011), Pobbe et al. (2012), and Leonzino et al. (2019)
*Pak2*^+/−^	• Repetitive self-grooming behavior • Increased marble-burying behavior	• Changes in striatal dendritic spines • Reduced spine density in cortex and hippocampus • Impaired LTP in CA1 hippocampal region • Reduced actin polymerization and perturbation of actin network		Wang Y. et al. (2018)
*Pcdh19 X*^LacZ^/Y	• Repetitive grooming behavior • Increased rearing behavior	• Impaired migration and dendritic arborization of hippocampal CA1 neurons • Decreased GABA-A receptor surface expression and transmission		Bassani et al. (2018) and Lim et al. (2019)
*Pten*^+/−^	• Repetitive digging and increased marble-burying behavior • Reduced sensorimotor gating • Increased depression-like behavior	• Increased mTOR signaling • Alterations in the serotonin system • Altered synaptic scaffolding proteins (PSD-95, sapap1, sap-102)		Page et al. (2009), Clipperton-Allen and Page (2014, 2015), Lugo et al. (2014), and Rademacher and Eickholt (2019)
		• Decreased mGluR in the hippocampus • Structural aberrations in Purkinje cells dendrites and axons		
*PV*^−/−^	• Higher-order reversal learning in T-maze	• Decreased parvalbumin levels • Altered excitatory and inhibitory synaptic transmission • Decreased inhibition of pyramidal neuron output • Loss of inhibitory synapses resulting in hyperexcitation of cortical circuits • Reduced cortical volume, increased cerebellar volume	• 17-beta estradiol	Filice et al. (2018)
*Sapap3*^−/−^	• Compulsive self-grooming	• Glutamatergic transmission defects at cortico-striatal synapses • Elevated mGluR5 signaling	• Sapap3 re-expression in the striatum • Optogenetic stimulation of the lateral orbitofrontal cortex • mGluR5 inhibition • Serotonin uptake inhibitor (fluoxetine)	Welch et al. (2007), Bienvenu et al. (2009), and Burguière et al. (2013)
*Scn1a*^+/−^	• Repetitive self-grooming and circling • Hyperactivity	• Increased PFC excitation • Altered GABAergic activity in PFC		Han et al. (2012)
*Shank1*^+/−^, *Shank1*^−/−^	• Repetitive self-grooming • increased acquisition of spatial memory • motor deficits • mild anxiety-like phenotype • Reduced exploratory locomotion	• A decrease in mEPSC, altered glutamatergic synapse • Altered maturation of postsynaptic dendritic spines • Reduced density of CA1 pyramidal neurons dendritic spines		Hung et al. (2008), Silverman et al. (2011), Sungur et al. (2014), and Sala et al. (2015)
*Shank2*^−/–^ (*exon 7 deletion*)	• Repetitive grooming • Hyperactivity • Anxiety-like behavior • Increased locomotor activity	• Increased NMDAR-dependent LTP and altered NMDAR-mediated synaptic transmission • Reduced spine density • Increased levels of GluN2A, GluN1, GluN2B, GluA2 glutamate receptor subunits in hippocampus and striatum		Schmeisser et al. (2012)
*Shank2* (*exons 6, 7 deletions and frameshift affecting both splice variants Shank2a and Shank2b*)	• Stereotypic jumping • Impaired spatial learning and memory (Morris water maze) • Impaired nesting behavior • Hyperactivity • Anxiety-like behavior • Increased grooming in the novel object recognition area	• Reduced activity of glutamatergic NMDA receptors • Impaired LTP and LTD at Schaffer-collateral-CA1-pyramidal (SC-CA1) synapses • Reduced NMDA/AMPA ratio at SC-CA1 synapses • Decreased NMDAR-mediated synaptic transmission		Won et al. (2012)
*Shank3* (*exon 21 deletions including Homer binding domain*)	• Repetitive grooming in older mice • A deficit in spatial learning and memory • Impaired motor coordination • Aberrant locomotor response to novelty • Increased novel object avoidance (in marble-burying test)	• Decreased excitatory postsynaptic NMDA/AMPA current ratio in the hippocampal CA1 region • Reduced LTP in CA1 hippocampus • Increased mGluR5 levels in synaptic fractions		Kouser et al. (2013)
*Shank3*^e4^^–^^22^ (*exons* 4–22 *deletion*)	• Excessive Repetitive self-grooming • Reduced locomotion • Deficient motor performance • Anxiety-like behavior • Impaired striatal learning	• Impaired postsynaptic SAPAP, mGluR5-Homer scaffolding proteins, and mGluR5 signaling in striatal neurons • Impaired striatal LTD and synaptic plasticity • Decreased neurotransmission in corticostriatal circuits • Reduced striatal spine density	• mGluR5 antagonist (MPEP)	Wang X. et al. (2016)
*Shank3A^e4^^–^^9^* *heterozygous and knockout (exons 4–9 deletion encoding ANK domain)*	• Repetitive self-grooming • An enhanced head pokes (hole board test) • Mild motor abnormalities including difficulty in motor coordination in KO mice • Motor learning deficits in KO mice • Impaired novel and spatial object recognition learning and memory	• Reduced Homer1b/c, GKAP, and AMPAR subunit GluA1, GluA2, GluA3 levels at PSD in KO mice indicating altered synaptic scaffolding proteins and receptor subunits • Reduced spine density and increased spine length in CA1 hippocampus • Impaired hippocampal LTP (in both KO and HTZ), glutamatergic synaptic transmission, and synaptic plasticity in knockout mice • Reduced NMDA/AMPA ratio at excitatory synapses onto striatal MSNs (in both KO and HTZ)		Bozdagi et al. (2010), Wang et al. (2011), Yang et al. (2012), Drapeau et al. (2014), and Jaramillo et al. (2016)
*Shank3b*^−/–^	• Repetitive self-grooming • Attention-deficit	• Functionally impaired AMPA and NMDA receptors • Decreased D2 MSNs AMPA receptor responses • Deficits of hippocampal synaptic plasticity and its association with the impaired remodeling of the actin cytoskeleton	• Enhancing the activity of the indirect striatopallidal pathway • Subthalamic nucleus stimulation • Partial 5-HT1A receptor agonist (tandospirone) in Shank3B^+/–^	Bozdagi et al. (2010), Peça et al. (2011), Wang et al. (2011), Schmeisser et al. (2012), Duffney et al. (2013), Sala et al. (2015), Chang et al. (2016), Peixoto et al. (2016), Harony-Nicolas et al. (2017), and Dunn et al. (2020)
*Shank3B*^−/–^ (*PDZ domain deletion*)	• Excessive and self-injurious self-grooming • Anxiety-like behavior	• Reduced levels of synaptic scaffolding proteins SAPAP3, Homer-1b/c, PSD93 and glutamate receptor subunits GluR2, NR2A, and NR2B at PSD • Neuronal hypertrophy • Reduced dendritic spine density		Peça et al. (2011)
		• Increased caudate volume • Decreased C-S circuits neurotransmission		
*Sh3rf2*^+/–^	• Increased jumping and rearing behavior • Increased marble burying and digging • Hyperactivity	• Abnormal dendritic spine development in the hippocampus • Changes in the composition of glutamate receptor subunits NR2A and GluR2 • Altered AMPA receptor-mediated synaptic transmission in CA1 hippocampus		Wang S. et al. (2018)
*Tsc2f*/−;Cre (Tsc2 deletion in cerebellar Purkinje cells)	• Increase marble-burying	• Cerebellar GABAergic Purkinje cell loss • Abnormalities in axonal pathfinding		Reith et al. (2013)
*Ube3A*^m-/p+^	• Decrease marble burying and rearing • Reversal learning deficit (MWM) • Impaired motor coordination	• Reduced mGluR-LTD • Altered mGluR signaling • Changes in calcium-dependent CAMKII activity in the hippocampus		Weeber et al. (2003), Huang et al. (2013), and Pignatelli et al. (2014)
*VPA*	• Repetitive self-grooming • Marble burying • Decrease pre-pulse inhibition • Reduced social behaviors	• Increased glutamatergic excitatory signaling • Hyperexcitable local connectivity • A decrease in parvalbumin-positive inhibitory interneurons • Elevated brain serotonin levels • Apical dendritic arborization complexity • Decreased PTEN expression and increased p-AKT protein levels in hippocampus and cortex	• mGluR5 receptor antagonist, MPEP • Environmental enrichment • Betaine (methyl group donor in homocysteine metabolism, prevents homocysteine accumulation) • NMDA receptor antagonist (agmatine)	Schneider and Przewłocki (2005), Schneider et al. (2006), Rinaldi et al. (2007), Tsujino et al. (2007), Snow et al. (2008), Mehta et al. (2011), Choi et al. (2016), Kim et al. (2017), Mahmood et al. (2018), and Huang et al. (2019)
*16p11*^+/–^	• Repetitive circling and climbing • Hyperactivity • Increased locomotion	• Increased dopamine D2 receptor-expressing striatal neurons • Decreased dopamine D2 receptor-expressing cortical neurons • Synaptic function defects • Volumetric alterations in striatum, hypothalamus, and midbrain area		Horev et al. (2011) and Portmann et al. (2014)
*5Ko* (deletion of 5 kainate receptor subunits)	• Elevated self-grooming • Increased marble burying and digging • Increased perseverative behavior (Y-maze) • Motor problems	• Impaired corticostriatal synaptic transmission in the dorsal striatum • Altered NMDA/AMPA ratio • Reduced mEPSC frequencies • Reduced spine density of spiny projections neurons in the dorsal striatum		Xu et al. (2017)

*Treatment strategies discussed are from preclinical studies in rodent models targeting behavioral abnormalities including stereotypic behaviors*.

Fragile X syndrome (FXS) is caused by an expansion of a single trinucleotide sequence (CGG) resulting in silencing of FMR1, an X-linked gene coding for fragile X mental retardation protein (FMRP). FMR-1 protein, an RNA binding protein plays an important role in regulating synaptic proteins *via* mRNA translation and the development of neural synapses. In addition to mRNA binding, FMRP protein has diverse functions including protein-protein interactions, DNA damage repair *via* chromatin binding, regulation of Ca^2+^ signaling, and neuronal excitation/inhibition balance (Brown et al., 2010; Alpatov et al., 2014; Davis and Broadie, 2017; Filippini et al., 2017; Zhou et al., 2017). Hence, failure to express the FMR-1 protein results in the development of autistic symptoms such as repetitive and restricted behavior (Turner et al., 1996; Mazzocco et al., 1998; Spencer et al., 2005). Fragile X mutant models exhibit increased marble burying (Thomas et al., 2012; Gandhi et al., 2014), resistance to change in an operant task (Moon et al., 2006), learning deficits on water maze task, hyperactivity, anxiety, and inadequate pre-pulse inhibition of acoustic startle (D’Hooge et al., 1997; Peier et al., 2000; Spencer et al., 2005; Lauterborn et al., 2007; Errijgers et al., 2008). Fmr-1 null mice exhibit altered spine density and morphology on apical dendrites of occipital cortical layer 5 pyramidal cells (Comery et al., 1997; Beckel-Mitchener and Greenough, 2004). Also, Fmr1 knockout mice exhibit dysfunctional cortico-striatal circuitry, reduced long-term potentiation (LTP), and decrease in levels of synaptic proteins like NMDAR subunits NR1, NR2A, and NR2B in the medial prefrontal cortex (Lauterborn et al., 2007; Krueger et al., 2011; Zerbi et al., 2018). Gene therapy using human *FRM1* alleviates the low pre-pulse inhibition, hyperactivity, and anxiety behaviors in Fmr1-KO mice (Peier et al., 2000; Paylor et al., 2008; Spencer et al., 2008; Gholizadeh et al., 2014). Application of brain-derived neurotrophic factor (BDNF), mGluR5 antagonists, anti-purinergic therapy (suramin), minocycline, phosphodiesterase-4D negative allosteric modulator (BPN14770) and PI3K antagonist [GSK2702926A (GSK6A)] attenuates dendritic spine development aberrations, LTP impairments, and behavioral abnormalities in *Fmr1* mutant mice (Dölen et al., 2007; Lauterborn et al., 2007; Dölen and Bear, 2008; Bilousova et al., 2009; Naviaux et al., 2015; Gurney et al., 2017; Yau et al., 2018; Gross et al., 2019).

Angelman syndrome involves chromosome 15 deletions, particularly the q11–13 region, comprising the GABA_A_ receptor beta 3 subunit (GABRB3) and ubiquitin ligase (UBE3A) genes. GABRB3 and UBE3A genes play a role in regulating protein synthesis and synaptic plasticity (Weeber et al., 2003; Moy et al., 2006; Mardirossian et al., 2009). Mouse models of GABRB3 and UBE3A deletions exhibit ASD phenotype including developmental delay, hyperactivity, epilepsy, impaired motor function, learning deficits, and anxiety-related behaviors (DeLorey et al., 1998; Jiang et al., 2010; Tanaka et al., 2012). Mice with a mutation in Ube3A^m−/p+^ (maternal null mutation) exhibit deficits in LTP and changes in calcium-dependent CaMKII activity in the hippocampus (Weeber et al., 2003). The Ube3A^m^−/p+ mice show decreased marble burying, rearing behavior, and reversal-learning deficits in the Morris water maze (MWM) (Huang et al., 2013). Additionally, Gabrb3 deletions cause neuronal dysfunction *via* alterations in protein synthesis and GABA-A receptor-mediated synaptic transmission. The Gabrb3^−/−^ mice also exhibit repetitive circling behavior (Mercer et al., 2016; Orefice et al., 2016).

Another condition, tuberous sclerosis (TSC), involves mutation of either TSC1 and TSC2 genes that codes for proteins hamartin and tuberin, which act as tumor suppressors that regulate cell growth and the mTORC1 complex (Astrinidis and Henske, 2005; Inoki et al., 2005; Curatolo and Bombardieri, 2007). mTOR is a crucial part of signaling pathways involved in cell growth, protein synthesis, and axon formation (Choi et al., 2008; Huang and Manning, 2008). Tsc2^+/–^ mice with heterozygous TSC2 gene mutations exhibit learning, and memory deficits associated with aberrant mTOR signaling mediated LTP in the hippocampal CA1 region (Ehninger et al., 2008). Mice with *Tsc2* loss in cerebellar Purkinje cells (Tsc2f/−; Cre mice) display ASD-like behaviors, including social deficits and repetitive behavior (Reith et al., 2013). Further, Tsc2 mutant mice with *Tsc2* gene deletion from radial glial progenitor cells exhibit lamination aberrations, enlargement of neurons and glia, myelination defects, and astrocytosis (Way et al., 2009). Also, mice with ablated TSC1 expression in neurons show seizures and neuropathological aberrations including enlarged, ectopic neurons in the hippocampus, cortical, thalamic brain areas, alterations in glutamatergic synapses, abnormalities in cortical lamination, cytoskeleton, dendritic spine structure, and myelination (Tavazoie et al., 2005; Meikle et al., 2007). Application of mTORC1 inhibitors rapamycin and RAD001 [40-O-(2-hydroxyethyl)-rapamycin] ameliorates synaptic, cognitive, and behavioral deficits in a mouse model of tuberous sclerosis (Ehninger et al., 2008; Meikle et al., 2008; Zeng et al., 2008; Ehninger and Silva, 2011; Bateup et al., 2013).

Rett syndrome (RTT) is caused by mutations in the MECP2 gene located on the X-chromosome, which encodes for methyl-CpG-binding protein 2 (MeCP2) and affects brain development mostly in females (Ghidoni, 2007). Several mouse models of autism have been developed to study the effects of MeCP2 mutations (Chahrour and Zoghbi, 2007; Samaco et al., 2008). Mutant mice with truncated MeCP2 protein show repeated forelimb motions similar to repetitive hand movements in individuals with Rett syndrome (Table 1) (Shahbazian et al., 2002; Moretti et al., 2005). Dopaminergic deficits are implicated in RTT, such as decreased levels of dopamine transporter (DAT) (Wong et al., 1998), the altered density of dopamine D2 receptors in the striatum (Chiron et al., 1993), and reduced levels of tyrosine hydroxylase (TH), dopamine synthetic enzyme, in the striatum (Panayotis et al., 2011), suggesting striatal dysfunction in RTT individuals. Additionally, MeCP2 null mice exhibit deficits in motor coordination and motor learning along with memory deficits in the MWM. Environmental enrichment alters excitatory synaptic density in cortex and cerebellum, LTP deficit, increased BDNF levels in cortex, and rescued motor learning deficits (Lonetti et al., 2010).

Autism susceptibility genes, such as neuroligin genes (NL1, 2, 3, 4) encode the eponymous members of postsynaptic cell surface adhesion proteins that are crucial for synapse formation and maintenance (Südhof, 2008). Deletion and point mutation of neuroligin-3 (NL3) are associated with autistic behavioral phenotypes (Jamain et al., 2003; Levy et al., 2011). Overexpression of neuroligin-2 (NL2) in PFC leads to repetitive jumping behavior in mice (Table 1) (Hines et al., 2008). Moreover, deficits in neurexins, which are presynaptic cell adhesion proteins that serve as ligands for neuroligins and modulates synapse differentiation and maturation, control transmitter release, result in stereotypic grooming and altered nest-building behaviors in neurexin1a mutant mice (Etherton et al., 2009; Li and Pozzo-Miller, 2020).

**Figure 2:**
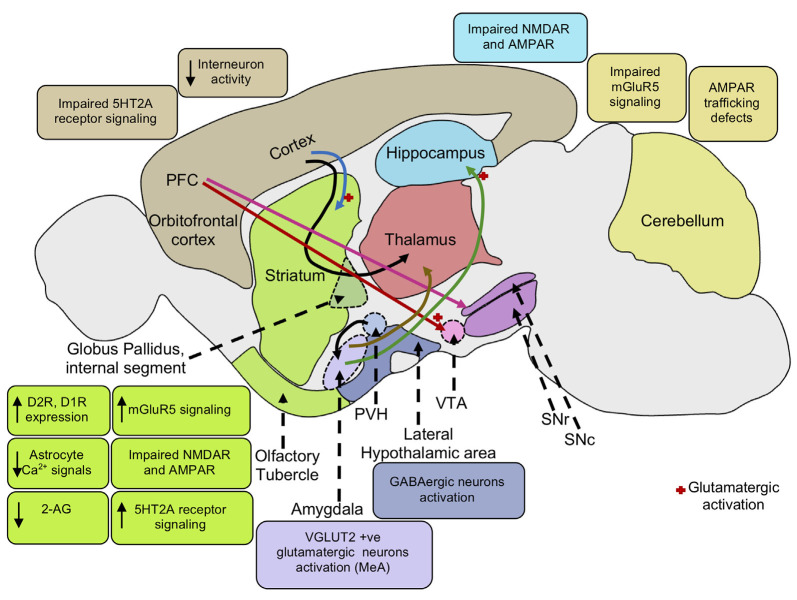
Neural mechanisms underlying repetitive behaviors. Increased mGluR5 signaling activates the striatal direct pathway leading to heightened motor cortex activity inducing repetitive behaviors. Impaired NMDA and AMPA receptors in the striatum and hippocampus also mediates stereotypic behaviors. Cortico-striatal and PFC-VTA glutamatergic projections induce repetitive behavior. PFC projections to the SNc causes striatal dopaminergic release promoting movement. The decrease in interneuron activity in the cortex and increase in dopamine D2, D1 receptor expression in the striatum leads to reduced GABAergic signaling in the cortex, enhancing motor cortical activity, and repetitive behaviors. Elevation of serotonin 5HT_2A_ receptor signaling in the dorsomedial striatum gives rise to stereotypic behaviors. Activation of VGLUT-positive glutamatergic neurons in the amygdala nucleus, MeA also results in stereotypic behaviors. Activation of glutamatergic projection from BLA to the ventral hippocampus leads to an increase in locomotor activity. Further, activation of lateral hypothalamic GABAergic neurons mediates an increase in locomotor activity and repetitive behaviors. Reduction in endocannabinoid 2-AG signaling in the striatum leads to an increase in glutamatergic output, enhancing motor cortex activity resulting in repetitive behaviors. Low astrocytic Ca^2+^ signals in the striatum elevate membrane GAT-3 expression that modulates striatal MSN activity *via* reduced ambient GABA levels inducing repetitive behavior. mGluR5, metabotropic glutamate receptor 5; NMDA, *N*-Methyl-d-aspartate; AMPA, α-amino-3-hydroxy-5-methyl-4-isoxazolepropionic acid; PFC, prefrontal cortex; VTA, ventral tegmental area; SNc, substantia nigra pars compacta; SNr, substantia nigra pars reticulata; PVH, paraventricular nucleus of the hypothalamus; GABA, gamma-aminobutyric acid; D2R, dopamine receptor D2; D1R, dopamine receptor D1; 5HT_2A_, 5-hydroxy-tryptamine receptor 2A subtype; VGAT, vesicular GABA transporter; MeA, medial nucleus of the amygdala; BLA, basolateral amygdala; 2-AG, 2-arachidonoyl glycerol; GAT-3, GABA transporter 3; MSN, and medium spiny neuron.

SH3 and multiple ankyrin repeat domains 1, 2, and 3 (SHANK1, SHANK2, and SHANK3) are postsynaptic scaffolding proteins present in excitatory synapses that are important for synaptic development and function (Grabrucker et al., 2011; Guilmatre et al., 2014). The Shank3 protein contains multiple conserved motifs, comprising an ANK repeat, PDZ, and SAM domains, a proline-rich cluster, and SH3 (Gundelfinger et al., 2006; Kreienkamp, 2008). The SHANK proteins also regulate spine morphology and receptor endocytosis, promote interaction of signaling pathways and facilitate synaptic plasticity, crucial for the process of learning and memory (Ehlers, 1999; Sheng and Kim, 2000; Monteiro and Feng, 2017). Mutations in Shank genes are implicated in ASD (Schmeisser, 2015). In particular, PMS or 22q13.3 deletion syndrome is characterized by developmental and speech delays, intellectual disability, reduced motor function, and ASD. PMS is caused by loss of function of the *SHANK3* gene resulting in reduced expression of SHANK3 protein, affecting synaptic transmission and plasticity (Costales and Kolevzon, 2015). SH3 and multiple ankyrin repeat domains 3b mutant mice (Shank3b^−/−^) show repetitive grooming behavior (Table 1) (Peça et al., 2011; Schmeisser et al., 2012). Moreover, Shank3B mutant mice manifest functionally impaired AMPA and NMDA receptors (Peça et al., 2011; Sala et al., 2015; Peixoto et al., 2016) (Figure 2). Shank1^+/−^ mice display increased self-grooming behavior during adulthood (Sungur et al., 2014), while Shank2^−/−^ mice manifest hyperactivity and repetitive jumping behavior along with the reduced activity of NMDA receptors (Table 1) (Schmeisser et al., 2012; Won et al., 2012). In contrast, Shank1 genotypes (Shank1^+/+^, Shank1^+/–^ and Shank1^−/−^) exhibit high self-grooming behaviors, but which are confounded by behavioral testing or housing conditions. Shank1 null mutant mice show decreased transitions in the light-dark test, suggesting anxiety-related phenotypes and reduced motor abilities (Silverman et al., 2011).

Contactin associated protein-like 2 (CASPR-2) transmembrane protein is encoded by the CNTNAP2 gene of the neurexin superfamily that primarily mediates cell-cell adhesions in the nervous system (Rodenas-Cuadrado et al., 2014). Also, the CNTNAP2 gene plays an important role in the formation of dendritic spines and dendritic arborization (Anderson et al., 2012). Cntnap2 KO mice exhibit neuronal migration abnormalities, decreased cortical interneurons number, and aberrant hippocampal and cortical network activity (Peñagarikano et al., 2011). Also, the Cntnap2 mutant mice show reduced densities of dendritic spines along with decreased levels of AMPA receptors (AMPAR) subunit GluA1 in the spines (Gdalyahu et al., 2015; Varea et al., 2015; Gao et al., 2019). Further, the decreased number of parvalbumin-positive interneurons in the striatum results in altered activity of the cortico-striatal-thalamic pathway underlying repetitive behaviors (Lauber et al., 2018). Mice with the CNTNAP2 mutation display repetitive self-grooming behavior, rescued by risperidone, a dopamine D2 receptor antagonist (Table 1) (Peñagarikano et al., 2011), thereby, decreasing dopaminergic function and cortical activation (Parr-Brownlie and Hyland, 2005).

In addition to the above autism susceptibility genes, many other genes implicated in autistic phenotypes have been investigated in preclinical studies. Mutations in protocadherin 19 (*PCDH19*) chromosome X-linked gene, leads to Epilepsy in Females with Mental Retardation (EFMR) disease, cognitive impairments, and autistic phenotype (Ryan et al., 1997; Dibbens et al., 2008; Hynes et al., 2010; Specchio et al., 2011). *PCDH19* gene encodes PCDH19 protein which is a cell-adhesion protein. PCDH19 regulates hippocampal neurons maturation, migration, and GABAergic transmission *via* binding with GABA-A receptor alpha subunit (Bassani et al., 2018). Additionally, PCDH19 interacts with intracellular protein NONO, involved in the modulation of steroid hormone receptors (Pham et al., 2017). Male mice with *Pcdh19* knockout (*Pcdh19* X^*LacZ*^/Y) exhibit increased rearing and stereotypic grooming behaviors (Lim et al., 2019).

Ephrins are membrane-bound proteins acting as ligands of ephrin receptors, belonging to receptor tyrosine kinases (RTKs) family which are transmembrane proteins. They serve important functions including angiogenesis, axon guidance, cell migration, tissue border formation, and synaptic plasticity (Chin-Sang et al., 1999; Kullander and Klein, 2002; Martínez and Soriano, 2005; Héroult et al., 2006; Aoto and Chen, 2007; Klein, 2009). In CNS, ephrins and Eph receptors are involved in axon pathfinding, topographic development of different brain regions and connectivity, neuronal migration, dendritic spine maturation, synapse formation, and plasticity (Gao et al., 1996; Dalva et al., 2000; Ethell et al., 2001; Grunwald et al., 2001, 2004; Henkemeyer et al., 2003; Murai et al., 2003; Palmer and Klein, 2003; Bolz et al., 2004; Klein, 2004; Yamaguchi and Pasquale, 2004; Egea and Klein, 2007; Akaneya et al., 2010; Triplett and Feldheim, 2012). Deletion of ephrin-A2 in mice exhibits impairment of behavioral flexibility in visual discrimination reversal-learning task (Arnall et al., 2010). Mice with a double knockout of ephrin-A2 and ephrin-A3 manifest excessive stereotypic facial grooming behaviors, resulting in face lesions. Also, they show reduced locomotor activity, shift towards grooming in the marble-burying assay, and increased pre-pulse inhibition of acoustic startle (Wurzman et al., 2015). The repetitive grooming behavior in double knockout mice suggests abnormalities in sensorimotor gating (Ben-Sasson et al., 2007; Perry et al., 2007; Wurzman et al., 2015). Ephrin-A2 and ephrin-A3 are located at excitatory synapses in multiple brain regions. Their deletions may result in altered excitability of forebrain networks suggesting defective processing of sensory information (Qiu et al., 2012; Wurzman et al., 2015).

Phosphoinositide signaling is important for cell survival and proliferation. Phosphoinositide 3-kinase (PI3K), Akt (serine/threonine kinase), and mammalian target of rapamycin (mTOR) are important interlinks in the PI3K pathway and are activated by upstream receptor tyrosine kinases (RTKs) and regulates protein synthesis for cell growth and proliferation (Cantley, 2002). Phosphatase and tensin homolog

deleted on chromosome 10 (PTEN), a tumor suppressor gene is a negative regulator of the PI3K/AKT/mTOR signaling pathway (Ali et al., 1999; Sansal and Sellers, 2004). *Pten* is an ASD candidate risk gene and its mutation is reported in a subset of autistic cases with macrocephaly (Butler et al., 2005; Herman et al., 2007; Varga et al., 2009). Mice with PTEN deletions in cortical and hippocampal neurons show macrocephaly and ASD behavioral deficits, including seizures, increased anxiety, and learning deficits. The conditional *Pten* mutant mice exhibit neuronal hypertrophy associated with abnormal activation of the Akt/mTOR pathway and Gsk3b inactivation (Kwon et al., 2006). Additionally, conditional *Pten* knockout in astrocytes results in increases in their size (Fraser et al., 2004). Further, *Pten* conditional KO mice exhibit increased spine number, myelination defects, and changes in synaptic structure and transmission (Fraser et al., 2008). Germline *Pten*^+/–^ male mice also exhibit increased marble burying and digging, suggesting repetitive behavioral phenotype (Clipperton-Allen and Page, 2014, 2015). Deletion of PTEN causes changes in synaptic scaffolding proteins (PSD-95, Sapap1, sap-102) and reduced mGluR expression in the hippocampus (Lugo et al., 2014). PTEN also exhibits critical functions during development, with significant implications for autism and neurodevelopmental disorders (Rademacher and Eickholt, 2019). Hence, PTEN dysfunction in neurons has profound effects on neuronal morphology and connectivity resulting in ASD-like behaviors.

Additionally, the Homeobox protein (Hoxb8) protein is encoded by the HOXB8 gene, a member of the homeobox-containing group of transcription factors, involved in developmental processes such as positioning along the anterior-posterior axis and other physiological functions. *Hoxb8* mutant mice display excessive grooming behavior resulting in skin lesions and anxiety-like behavior (Greer and Capecchi, 2002). In mouse brains, Hoxb8 cell lineage is present in the microglia. *Hoxb*8 mutant mice with Hoxb8 mutations in microglia, exhibit increased cortical dendritic spine density and dendritic spines in the striatum, defects in synapse structure, LTP, and miniature postsynaptic currents. Long-term application of fluoxetine (SSRI) attenuates excessive grooming and hyperactivity in *Hoxb8* mutant mice. Hence, Hoxb8 in microglia may play role in the modulation of cortico-striatal circuits and associated grooming behavior (Chen et al., 2010; Nagarajan et al., 2018).

KCNQ/K_v_7 channels mediate voltage-dependent outward potassium currents regulating resting membrane potential and decreasing neuronal excitability. KCNQ2 encodes subunits of neuronal KCNQ/K_V_7- K^+^ channels, K_V_7.2, which are present in the hippocampus and cortex. Mutations in K_V_7.2 are associated with developmental delay and autism (Cooper et al., 2001; Yue and Yaari, 2006; Shah et al., 2008; Brown and Passmore, 2009). Mice with heterozygous null mutations in the KCNQ2 gene (KCNQ2^+/−^) exhibit elevated locomotor activity, hyperactivity, exploratory and repetitive grooming, suggesting loss of K_V_7.2 is linked to ASD behavioral abnormalities (Kim et al., 2020).

Kin of Irregular Chiasm-like 3 (*KIRREL3*) gene mutations are linked with neurodevelopmental disorders including autism and intellectual disability (Bhalla et al., 2008; Iossifov et al., 2012; Baig et al., 2017). The KIRREL3 gene encodes the Kin of IRRE-like protein 1 (KIRREL3), also called NEPH2 (Sellin et al., 2003). KIRREL3 (NEPH2) is a member of the KIRREL protein family of transmembrane proteins that includes KIRREL (NEPH1) and KIRREL2 (NEPH3). KIRREL3 plays a role in kidney blood filtration function and is a synaptic cell-cell adhesion molecule (Gerke et al., 2006; Neumann-Haefelin et al., 2010). Kirrel3 in mice is present in the developing cochlea, retina, and olfactory neuroepithelial regions and in the adult nervous system comprising sensory regions (Morikawa et al., 2007). Disruption of the function of the KIRREL3 gene is associated with alterations in brain function. The gene is implicated in neural circuit development including neuronal migration, axonal fasciculation, and synapse formation (Serizawa et al., 2006; Nishida et al., 2011; Prince et al., 2013). KIRREL3 gene knockout in mice leads to alterations in synapses connecting dentate gyrus (DG) neurons to GABAergic neurons but no changes were observed in synapses linking DG neurons to CA3 neurons. This resulted in the disruption of DG synaptic activity and overactivation of CA3 neurons (Martin et al., 2015). KIRREL3 KO mice display increased rearing repetitive behavior, hyperactivity, impaired novel object recognition, and sensory abnormalities (Choi et al., 2015; Hisaoka et al., 2018).

Furthermore, Integrin-beta3 gene encodes integrin beta-3 protein which is a cell-surface protein (a member of alpha/beta heterodimeric receptors) and is involved in various functions including cell adhesion/migration, cell-extracellular matrix interactions, and axon/dendrite outgrowth (Sosnoski et al., 1988; De Arcangelis and Georges-Labouesse, 2000; Clegg et al., 2003). Increased integrin-beta3 activity leads to elevated SERT transport of 5-HT and increased blood serotonin levels which are reported in autistic individuals (Carneiro et al., 2008). Mice with a mutation in the integrin-beta3 gene exhibit elevated grooming in novel environments with no changes in activity in the open field test. Disruption of integrin-beta3 protein impairs platelet aggregation resulting in increased bleeding times and hemorrhages. Additional studies are required to ascertain behavioral abnormalities in integrin-beta3 deficient mice (Carter et al., 2011).

Netrin-G ligand 2 (NGL-2)/LRRC4 is a leucine-rich repeat comprising postsynaptic cell adhesion molecule which interacts with PSD-95, excitatory postsynaptic scaffolding protein, and netrin-G2, a presynaptic cell adhesion molecule (Lin et al., 2003; Kim et al., 2006; Woo et al., 2009; Matsukawa et al., 2014). NGL-2 is implicated in intellectual disability and ASD (Jiang et al., 2013; Sangu et al., 2017). NGL-2 is involved in the regulation of glutamatergic synapse development and excitatory transmission (DeNardo et al., 2012). Mice with mutations in NGL-2 (*Lrrc4^−/−^*) exhibit reduced hippocampal NMDA receptor synaptic plasticity (Soto et al., 2013, 2018; Um et al., 2018). Lrrc4^−/−^ mice show repetitive self-grooming behavior which is rescued by D-cycloserine, the NMDAR agonist. Also, Lrrc4^−/−^ mice exhibit impaired spatial learning in the MWM test and mild anxiety-like behavior (Um et al., 2018).

Similarly, Nerve injury-induced protein 1 (Ninjurin1/Ninj1), is a cell-adhesion molecule involved in nerve regeneration, angiogenesis, inflammation, and cancer (Araki and Milbrandt, 1996; Ifergan et al., 2011; Matsuki et al., 2015; Jang et al., 2016). Ninj1 is expressed in cortico-thalamic circuits and is implicated in the regulation of synaptic transmission. Mutation in Ninjurin1 (Ninj1) in mice leads to excessive grooming to the point of inducing hair loss and lesions and increased anxiety-like behavior. Also, Ninj1 mutant mice exhibit glutamatergic alterations in the brain, including elevated ionotropic glutamate receptors synaptic expression and mEPSCs amplitude. Stereotypic grooming in these mice is alleviated by fluoxetine (SSRI), correlating with direct inhibitory effects of fluoxetine on NMDA receptors (Le et al., 2017).

SH3RF2 gene present in the 1.8 Mb microdeletion at 5q32 is implicated in autism (Gau et al., 2012; Yuen et al., 2017). It plays a role as an anti-apoptotic regulator of the JNK pathway *via* degrading SH3RF1 protein that activates the JNK pathway (Wilhelm et al., 2012; Kim et al., 2014). Mice with haploinsufficiency of Sh3rf2 (Sh3rf2^+/–^) show increased jumping, rearing behavior, bury more marbles in the marble-burying test correlating with elevated digging behavior and hyperactivity. Abnormalities in dendritic spine development in the hippocampus, AMPAR-mediated excitatory synaptic transmission in CA1 hippocampus, altered hippocampal pyramidal neurons membrane properties, and increases in NR2A and GluR2 glutamate receptor subunits in the hippocampus are observed in Sh3rf2^+/–^ mutant mice (Wang S. et al., 2018).

Additionally, the p21-activated kinase 2 (*PAK2*), a serine/threonine kinase, activated by Rho GTPases plays a crucial role in regulating cytoskeleton remodeling, dynamics, the formation of postsynaptic dendritic spines, and cortical neuronal migration (Bokoch, 2003; Boda et al., 2006; Asrar et al., 2009; Causeret et al., 2009; De La Torre-Ubieta et al., 2010). Mutations in the *PAK2* gene are implicated in ASD (Willatt et al., 2005; Quintero-Rivera et al., 2010; Sagar et al., 2013). Haploinsufficiency of Pak2 leads to reduced spine densities in cortex and hippocampus, impaired hippocampal CA1 LTP, decreased phosphorylation of actin regulators LIMK1, cofilin, and reduced actin polymerization. Pak2^+/–^ mice show repetitive grooming behavior and bury more marbles in the marble-burying test (Wang Y. et al., 2018). This suggests PAK2 is critical in brain development and its mutation contributes to autistic phenotypes.

The SCN1A gene heterozygous loss of function mutation results in Dravet Syndrome. Haploinsufficiency of the SCN1A gene affects the α subunit of the voltage-gated sodium channel (Na_V_1.1) in mice leading to autistic behavioral phenotypes, including hyperactivity and stereotypic behaviors such as self-grooming and circling behaviors. Scn1a^+/−^ mouse model of autism exhibit increased excitation in the prefrontal cortex (PFC). Deletion of sodium channels (Na_v_1.1) in cortical interneurons causes reduced sodium (Na^+^) currents and neurotransmission of GABAergic interneurons resulting in altered GABAergic activity, hyperexcitability, and behavioral impairments in the mutant mice (Table 1) (Han et al., 2012).

Mutations in receptor proteins are also involved in autistic phenotypes. Oxytocin is a peptide produced in the brain, particularly in the paraventricular nuclei and hypothalamic supraoptic. It is secreted primarily by the posterior pituitary gland into the circulation (Lee et al., 2009). Oxytocin facilitates biological effects by binding to the oxytocin receptor (Oxtr). The oxytocin receptor is mainly found in the amygdala, hippocampus, olfactory lobe, and hypothalamus areas of the brain (Gould and Zingg, 2003). *Oxtr*^−/−^ mice exhibit autistic-like phenotypes, increased self-grooming behavior in a visible burrow system (VBS) (Pobbe et al., 2012). *Oxtr*^−/−^ mice also exhibit cognitive inflexibility during the reversal phase in the T-maze test and increased aggression. *Oxtr*^−/−^ mice exhibit alterations in excitatory synaptic markers including PSD95, gephyrin scaffolding proteins, and glutamatergic, GABAergic receptors along with changes in striatal dendritic spines, indicating striatal dysfunction (Sala et al., 2011; Leonzino et al., 2019).

Environmentally induced alterations to the developing nervous system, such as through specific teratogenic agents or restricted housing also contribute to the etiology of ASD. *In utero* valproic acid (VPA), an antiepileptic drug, exposed mice and rats show increased repetitive behaviors, such as self-grooming along with reduced social interactions and communication dysfunction (Schneider and Przewłocki, 2005; Bromley et al., 2008).

C58/J, an inbred mice strain, show social deficits, repetitive backward somersaulting and hind limb jumping behaviors, restricted novel hole-board exploration, and reversal-learning deficits in the appetitive operant task (Moy et al., 2008b; Ryan et al., 2010; Muehlmann et al., 2012; Whitehouse et al., 2017). The hole-board test measures the number of nose-pokes (head-dipping) into holes in the floor arena as a measure of exploratory behavior (Moy et al., 2008a). Moreover, BTBR, an inbred mouse strain, shows ASD-like behavioral phenotype including social, communication deficits and stereotypic behaviors (McFarlane et al., 2008; Silverman et al., 2010; Wöhr et al., 2011). Balb/c mice, another inbred strain shows ASD-like behaviors, such as sociability deficits and stereotypic behaviors. Functional alterations in NMDAR mediated activity and elicitation of jumping and circling behavior by NMDAR antagonist MK-801 application is described in Balb/c strain (Deutsch et al., 1997; Burket et al., 2010).

Deer mice belong to a diverse *Peromyscus* genus of Cricetidae rodent family that is native to North America and utilized as a laboratory animal model for basic and applied research (Joyner et al., 1998; Crossland and Lewandowski, 2006). Deer mice exhibit repetitive behavior including hindlimb jumping and backward somersaulting upon being maintained in standard laboratory housing. The repetitive behaviors showed by deer mice occur at an increased rate, apparent during initial development and continuing across the lifespan. Deer mice also display reversal learning deficits in a procedural learning behavioral task involving learning to change spatial habits upon relocation of reinforcement in a T-maze (Hadley et al., 2006). Hence, deer mice are used as animal models of repetitive/restricted behaviors in autism (Powell et al., 2000; Lewis et al., 2007; Bechard et al., 2017).

## Glutamatergic and Gabaergic Signaling

The normal balance of excitation and inhibition (E/I) in the forebrain is maintained by excitatory glutamatergic neurons and inhibitory GABAergic interneurons. The major excitatory neurotransmitter in the cortex is glutamate, which activates two types of receptors, i.e., ionotropic and metabotropic G-protein coupled receptors (Mehta et al., 2011). Increased excitatory signaling, hyper-excitable local connectivity, and decreases in inhibitory interneurons accompany repetitive behavioral changes in the brains of ASD animals (Rinaldi et al., 2007; Gogolla et al., 2009). Interestingly, these behaviors are ameliorated by environmental enrichment, correlating to functional alterations in neural circuitry by modifying cortical excitatory and inhibitory synaptic density, LTP, increasing BDNF expression and synaptic plasticity in the cortical network (Schneider et al., 2006; Baroncelli et al., 2010; Lonetti et al., 2010; Reynolds et al., 2013; Jung and Herms, 2014).

Glutamatergic signaling plays a crucial role in the modulation of repetitive behaviors. On the one hand, NMDA receptors play important roles in the regulation of neurotransmitter release such as glutamate affecting excitatory neural pathways. For instance, intra-striatal injections of NMDA, glutamate receptor ligand, induces repetitive behaviors caused by elevated glutamatergic activity in the basal ganglia motor circuits (Karler et al., 1997). Deer mice exhibit repetitive behaviors, such as excessive jumping and backward flips, attenuated by interrupting cortico-striatal glutamatergic projections *via* striatal injection of NMDA receptor antagonist MK-801 (dizocilpine) (Presti et al., 2003). Mice with astrocyte-specific inducible deletion of GLT-1 (GLAST^CreERT2/+^/GLT1^flox/flox^, iKO) manifesting stereotypic grooming behavior is alleviated by memantine, NMDA receptor antagonist (Aida et al., 2015).

On the other hand, NMDA receptors are also expressed on the surface of GABAergic neurons modulating their inhibitory tone and controlling oscillations of pyramidal neurons involved in the regulation of neuronal rhythms and activity (Benes, 2010; Deutsch et al., 2010). For instance, systemic application of anti-glutamatergic agents, phencyclidine (PCP), an NMDA receptor antagonist, evokes stereotypic behaviors, including self-grooming in rodents. NMDA antagonist application might inhibit excitation of GABAergic inputs onto pyramidal neurons causing disinhibitory (i.e., hyperexcitation of pyramidal neurons) increase in glutamate efflux and glutamatergic neurotransmission *via* AMPA and non-NMDA receptors in the PFC, activating motor pathways (Liu and Moghaddam, 1995). This PCP or non-NMDA receptor-induced stereotypic grooming is alleviated by blocking AMPAR (non-NMDAR) mediated glutamatergic transmission between the prefrontal cortex (PFC) and ventral tegmental area (VTA) (Takahata and Moghaddam, 2003; Audet et al., 2006) (Figure 2). Also, neuroligin-1 (NL1) knockout mice exhibit a reduced NMDA/AMPA ratio in the dorsal striatum that correlates with repetitive grooming behavior, which is rescued by systemic administration of D-cycloserine, an NMDA receptor partial co-agonist (Blundell et al., 2010). Shank2^−/−^ mice manifest reduced NMDA receptor function and social deficits, normalized by application of D-cycloserine (Won et al., 2012). D-cycloserine is also revealed to improve sociability deficits and stereotypies in BTBR and Balb/c inbred mouse strains of ASDs (Deutsch et al., 1997, 2011a,b; Burket et al., 2013).

Dysfunction of glutamatergic signaling at the metabotropic glutamate receptor 5 (mGluR5) is implicated in neuropsychiatric disorders such as autism (Carlson, 2012) (Figure 2). As noted above, Fragile X Syndrome is a genetic disorder associated with autism and mental retardation. This disorder is caused by a loss of FMRP (Hagerman et al., 2017; Niu et al., 2017). The “mGluR theory of fragile X” suggests that FMRP and Group I metabotropic glutamate receptors (mGluRs) regulate protein synthesis at the synapse in an antagonist manner. mRNA translation at the synapse is activated by mGluRs and repressed by FMRP (Bear et al., 2004; Bear, 2005; Dölen and Bear, 2008). Fmr1-KO mice manifest increased expression of mGluR-dependent long-term depression (LTD) in the hippocampus, which is likely associated with alterations in mGluR signaling that contribute to repetitive behaviors in mutant mice (Table 1) (Yan et al., 2005; Nosyreva and Huber, 2006; Dölen and Bear, 2008; McNaughton et al., 2008; Pietropaolo et al., 2011). Also, Shank3^Δe4–22−/−^ mice (exons 4–22 deletion) exhibit excessive grooming and have reduced striatal postsynaptic mGluR5-Homer scaffolding proteins, altered mGluR5 signaling in the striatum and cortico-striatal circuit abnormalities (Wang X. et al., 2016). Interestingly, in the Ube3A^m^−/p+\ (maternal null mutation) mouse model of Angelman Syndrome, mGluR-dependent LTD and coupling of mGluR5 to Homer proteins in the hippocampus is enhanced (Pignatelli et al., 2014). A mouse model of Tuberous Sclerosis Tsc2^+/–^ exhibits reduced mGluR-LTD (LTD) in the hippocampus and altered levels of mGluR signaling Arc (activity-regulated cytoskeleton-associated) protein, which is crucial for AMPAR internalization in cerebellar LTD (Auerbach et al., 2011). This suggests that altered mGluR5 function may underlie cognitive and behavioral impairments in mutant mice models (Table 1) (Auerbach et al., 2011; Pignatelli et al., 2014).

**Figure 3:**
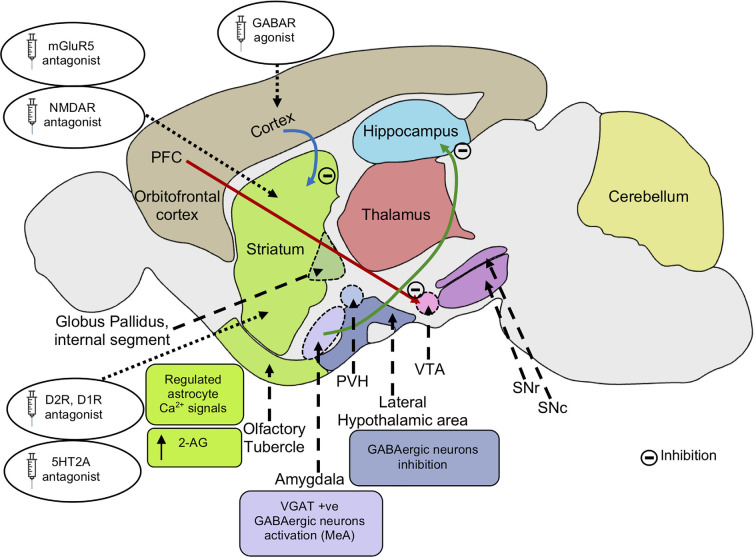
Possible mechanisms alleviating repetitive behaviors. Inhibition of mGluR5 signaling inhibits striatal direct pathway *via* suppressing dopamine D1 receptor signaling. The reduced D1R signaling results in decreased motor cortex activity. Inhibition of cortico-striatal and PFC-VTA glutamatergic projections alleviate repetitive behaviors. Application of GABA agonists in the cortex and dopamine D2R, D1R antagonist in the striatum leads to an increase in GABAergic signaling in the cortex, reducing motor cortical activity and repetitive behaviors. Application of serotonin 5HT_2A_ antagonist in the dorsomedial striatum also results in the rescue of repetitive behavior. Activation of VGAT-positive GABAergic neurons in the amygdala nucleus, MeA reduces repetitive behaviors. Inhibition of glutamatergic projection from BLA to the ventral hippocampus results in decreased locomotor activity. Inhibition of lateral hypothalamic GABAergic neurons leads to a decrease in locomotor activity and repetitive behaviors. Endocannabinoid 2-AG signaling in the striatum leads to reduced glutamatergic output, decreasing repetitive behaviors. Regulated astrocytes Ca^2+^ signals in the striatum modulate GAT-3 activity which maintains synaptic GABA levels, regulating striatal MSN activity and associated repetitive behavior. mGluR5, metabotropic glutamate receptor 5; NMDA, *N*-Methyl-d-aspartate; AMPA, α-amino-3-hydroxy -5-methyl-4-isoxazolepropionic acid; PFC, prefrontal cortex; VTA, ventral tegmental area; SNc, substantia nigra pars compacta; SNr, substantia nigra pars reticulata; PVH, paraventricular nucleus of the hypothalamus; GABA, gamma-aminobutyric acid; D2R, dopamine receptor D2; D1R, dopamine receptor D1; 5HT_2A_, 5-hydroxy-tryptamine receptor 2A subtype; VGAT, vesicular GABA transporter; MeA, medial nucleus of the amygdala; BLA, basolateral amygdala; 2-AG, 2-arachidonoyl glycerol; GAT-3, GABA transporter 3; MSN, the medium spiny neuron.

Several studies have demonstrated the therapeutic efficacy of the mGluR5 receptor antagonist, 2-methyl-6-phenyethyl-pyrididine (MPEP), on core behavioral deficits of autism. MPEP reduces repetitive and stereotypic behaviors in the VPA and BTBR mouse models of autism (Silverman et al., 2010; Mehta et al., 2011) (Figure 3). Additionally, MPEP application decreases marble burying stereotypic behavior in Fmr1 KO mice and excessive repetitive grooming in Shank3^Δe4–22−/−^ mice *via* modulation of mGluR5 signaling (Thomas et al., 2012; Gandhi et al., 2014; Wang X. et al., 2016). Also, in C58/J mice that exhibit stereotypic jumping behavior, backflips, and decreased exploratory behavior, blocking mGluR5 signaling *via* GRN-529, a mGluR5 negative allosteric modulator, rescues normal behavior (Silverman et al., 2012). The suppression of mGluR5 activity may modify NMDA receptor activity, since they are close associates at the postsynaptic density, suggesting NMDA receptor hyperfunction underlies jumping behavior in C58/J mice (Kim et al., 2016). Also, repetitive behavior and reversal learning deficits were attenuated by environmental enrichment in C58/J mice (Muehlmann et al., 2012; Whitehouse et al., 2017).

GABAergic signaling also plays a critical role in the regulation of stereotypic behaviors. For example, the application of GABA-enhancing drugs reduces self-grooming behavior in rodents (Silverman et al., 2015). Administration of R-baclofen, a selective GABA_B_ receptors agonist, alleviates repetitive self-grooming behavior in several ASD models, including the BTBR, Fragile X, C58/J, and idiopathic mice models (Han et al., 2014; Silverman et al., 2015). Also, the application of a GABA_A_ receptor-selective agonist, muscimol, into the bed nucleus of the stria terminalis (BNST) decreases self-grooming behavior induced by exposure to cat urine (Xu et al., 2012). Additionally, GABRB-3 knockout mice show hyperactivity and stereotypic behaviors such as circling (Moy et al., 2006). GABA also plays an important role in regulating stress and anxiety-related behaviors, with increased GABAergic signaling exerting anxiolytic effects and inhibition of stress and anxiety-induced grooming behaviors (Chao et al., 2010).

GABA receptor agonists regulate excitation and inhibition (E/I) balance, resulting in minimizing elevated excitation in motor cortical areas and parts of basal ganglia-thalamic circuitry (Lewis and Kim, 2009; Kim et al., 2016) (Figure 3). For instance, stereotypic behaviors evoked by amphetamine are diminished by the application of GABA receptor agonists (Lewis and Kim, 2009). Likewise, the application of GABA_A_ receptor antagonist, bicuculline, in the VTA enhances self-grooming in mice induced by alpha-melanocyte-stimulating hormone (MSH; De Barioglio et al., 1991). Also, muscimol injections into the substantia nigra pars reticulata (SNr) evoke repeated circling behavior in rats (Velíšek et al., 2005). Thus, altered GABA levels may modify basal ganglia activity by affecting dopaminergic neurons, leading to repetitive behaviors in rodents, as discussed further below (De Barioglio et al., 1991; Kim et al., 2016). Antidepressants/anxiolytics like fluvoxamine, bupropion, and diazepam alleviate repetitive digging behaviors (Hayashi et al., 2010). Moreover, Fmr1^−/−^ mice, discussed above, exhibit hyperexcitability due to reduced activity of fast-spiking interneurons (FSI) in the somatosensory and barrel cortex (Figure 2). GABA-receptor agonists decrease marble-burying behavior in these Fmr1 knockout mice (Draper et al., 2014). Hence, altered neural signaling and E/I balance underlie repetitive behaviors associated with ASD. Enhanced GABAergic function results in reduced cortical excitation and alleviates repetitive self-grooming behavior (Kalueff et al., 2016).

## Serotonergic Signaling

Serotonergic 5HT_2A_ receptors are found mainly in prefrontal cortical and striatal brain regions (Xu and Pandey, 2000), which are associated with repetitive behaviors in ASD (Di Martino et al., 2011; Langen et al., 2012; Delmonte et al., 2013). Differences in serotonergic components in the basal ganglia are associated with repetitive behaviors (Di Giovanni et al., 2006). For instance, deer mice exhibit a decreased density of serotonin transporters in the striatum (Wolmarans et al., 2013). And, injection of escitalopram, a selective serotonergic reuptake inhibitor (SSRI) alleviates some of the repetitive movements in deer mice, but with no effect on jumping behavior (Wolmarans et al., 2013). Additionally, optogenetic repetitive stimulation of the medial orbitofrontal cortex-ventromedial striatum pathway in mice leads to abnormal grooming behavior, which is rescued by fluoxetine administration, also an SSRI (Schmeisser et al., 2012). Family-based genetic association studies demonstrate linkages between serotonin transporter locus (SLC6A4) variants and rigid compulsive behavior (Sutcliffe et al., 2005), with the serotonin transporter gene (SLC6A4) subtype, 5HTTLPR, consistently associated with repetitive sensory and motor behaviors (Brune et al., 2006). Also, depleting tryptophan, a precursor of serotonin augments repetitive motor behaviors in autistic adults (McDougle et al., 1996).

Clinical and preclinical studies have implicated alterations in serotonin receptor activity, particularly 5HT_2A_ receptor signaling, in ASD symptomology (McBride et al., 1989; Veenstra-VanderWeele et al., 2012) (Figure 2). Systemic treatment with a serotonin 5HT_2A_ receptor antagonist decreases repetitive behaviors in the BTBR mouse model of autism, an inbred strain that shows similar ASD-like behavioral deficits to an idiopathic mouse model of autism (McFarlane et al., 2008; Amodeo et al., 2012, 2014, 2016). Further, infusion of M100907, a highly selective antagonist for 5HT_2A_ receptors into the dorsomedial striatum reduces grooming behavior and reversal learning deficits in BTBR mice. This regulation of reversal learning and grooming behavior by 5HT_2A_ receptor antagonist infusion into the dorsomedial striatum may be associated with a reduction in striatal direct pathway activation (Reiner and Anderson, 1990; Amodeo et al., 2017). However, 5HT_2A_ receptor antagonist infusion into the orbitofrontal cortex results in increased grooming behavior and perseveration in reversal learning (Amodeo et al., 2017). This altered grooming behavior by blocking of 5HT_2A_ receptor activity in the orbitofrontal cortex may be associated with an increased output by the orbitofrontal cortex *via* reduced interneuron activity, as the orbitofrontal infusion of GABA receptor agonist, muscimol, results in decreased grooming behavior in BTBR mice (Amodeo et al., 2017) (Figure 3).

Thus, elevated serotonin 5HT_2A_ receptor signaling in the dorsomedial striatum plays a critical role in the development of stereotyped behaviors, whereas normal 5HT_2A_ receptor activity in the orbitofrontal cortex contributes to attenuation of stereotyped behaviors in BTBR mice. Hence, abnormal serotonin receptor activity in various brain regions may contribute to restricted and repetitive behaviors.

## Dopaminergic Signaling and Basal Ganglia Circuitry

The cortico-basal ganglia-thalamic pathway implements motor patterned behaviors and is implicated in repetitive behaviors (Haber and Calzavara, 2009; Kalueff et al., 2016). Sequential patterns of behaviors, such as stereotyped sequential grooming movements, also called grooming chains, are carried out by these circuits in rodents (Berridge et al., 2005; Denys et al., 2013). Striatal lesions, particularly in the anterior dorsolateral region of the striatum, resulting in an inability to complete sequential grooming movements. Additionally, lesions of the ventral pallidum and globus pallidus result in disruption of grooming movements (Cromwell and Berridge, 1996), further underscoring their role in the regulation of complex and mechanistic sequenced behaviors.

Enhanced activity of basal ganglia circuitry results in increased hyperactivity and repetitive behaviors (Kim et al., 2015). In particular, the prefrontal cortical (PFC) projection to the substantia nigra pars compacta (SNc), leads to a dopaminergic release in the striatum, which promotes movement through opposing actions on direct and indirect basal ganglia pathways. Dopamine through D1 receptors is involved in the activation of the direct pathway, which in turn activates the motor cortex, resulting in movement. In contrast, dopamine through D2 receptors on neurons present in the indirect pathway, results in inhibition of the indirect pathway, also promoting movement (Gerfen et al., 1990; Gerfen, 1995). For example, amphetamine pretreated rats, when injected with a dopamine D2, D3 receptor antagonist, sulpiride, or the GABA antagonist, bicuculine, leads to repetitive behavior (Morency et al., 1985; Karler et al., 1998; Kiyatkin and Rebec, 1999). Further, these circuits are disrupted in autistic mouse models, which display PFC abnormalities. Namely, mice with mutations in the SCN1A gene leads to autistic-like phenotypes, including hyperactivity and stereotypic self-grooming and circling behaviors and increased excitation in the PFC (Han et al., 2012).

Dopamine plays a major role in modulating striatal pathways resulting in locomotion and repetitive motor behaviors. Application of Risperidone, which acts on different molecular receptors, including blocking of dopamine D2 receptors, leads to decreases in repetitive self-grooming behavior, perseveration, hyperactivity and rescues nesting deficits in Cntnap2^−/−^ mice. Similarly, systemic administration of haloperidol, a dopamine D2 receptor antagonist decreases motor cortex activity, thereby impeding locomotor movements in rats (Parr-Brownlie and Hyland, 2005). Interestingly, increased striatal dopamine D2 receptor expression leads to deficits in GABAergic activity, thereby enhancing prefrontal cortical (PFC) excitation (Li et al., 2011) (Figure 3). Hence, reduced repetitive and locomotory behavior caused by altered dopamine D2 receptor expression may be linked to heightened cortical GABAergic function and reduced PFC excitability.

Manipulation of the nigrostriatal dopamine pathway is sufficient for modulating many stereotyped behaviors (Lewis and Bodfish, 1998). Altered striatal dopamine activity is implicated in repetitive circling behaviors, which are observed in several mouse models of ASD (Vaccarino and Franklin, 1982; Ishiguro et al., 2007). Systemic administration of a dopamine precursor, L-DOPA, and a non-selective dopamine agonist, apomorphine into the striatum induces stereotyped behaviors in rodents (Ernst and Smelik, 1966; Presti et al., 2004). Likewise, injection of dopamine D1 receptor agonists evokes stereotypic and rigid behavioral phenotype in rodents (Berridge and Aldridge, 2000a, b). Furthermore, deer mice exhibit stereotyped behaviors, such as excessive jumping and backward flips, which are attenuated by intrastriatal injection of dopamine D1 receptor antagonist, SCH23390 (Presti et al., 2003) (Figure 3). Spontaneous motor stereotypies observed in deer mice exhibit a negative association with neuropeptide enkephalin expression, a marker of striatopallidal neurons, and is attenuated by combined administration of adenosine A2A receptor agonist CGS21680 and A1 receptor agonist CPA in a dose-dependent manner, indicating altered striatal pathway activity (Tanimura Y. et al., 2010). Environmental enrichment attenuates repetitive behavior by increasing activation through the indirect basal ganglia pathway, which also results in changes in dendritic spine density in the subthalamic nucleus (STN) and globus pallidus (GP) (Bechard et al., 2016).

Several ASD mice models exhibit alterations to dopaminergic nigrostriatal signaling. Mutant mice with heterozygous deletion of the syntenic region on chromosome 7F3 (16p11^+/–^) display decreased self-grooming behavior along with hyperactivity and increased stereotypic circling behavior. Neuroanatomically, these mice have increased numbers of dopamine D2 receptor-expressing neurons in the striatum, reduced number of cortical neurons manifesting dopamine D1 receptors, and synaptic function defects (Portmann et al., 2014) (Figure 2). Mice deficient in the DAT have elevated levels of dopamine and increased stereotypic sequential grooming behavior. Dopamine D1A receptor-deficient mice manifest disrupted and shorter duration grooming bouts (Cromwell et al., 1998). Neuroligin NL3 mutations result in a selective decrease of synaptic inhibition onto dopamine D1-expressing medium spiny neurons (MSNs) in the nucleus accumbens (NAc) and result in behavioral changes in mutant mice *via* reduced selective striatal synaptic function in the nucleus accumbens/ventral striatum (Rothwell et al., 2014). Apart from this, neuroligin-1 and 3 mutant mice show the abnormal function of dopamine D1 MSNs leading to autistic-like repetitive behaviors (Rothwell et al., 2014; Espinosa et al., 2015). In the Shank3 gene deletion mouse model, striatopallidal D2 MSNs show postsynaptic defects and decreased AMPAR responses (Mei et al., 2016; Zhou et al., 2016). Repetitive grooming in Shank3B mutant mice is rescued by enhancing indirect striatopallidal pathway activity (Wang et al., 2017). Additionally, synaptic plasticity is impaired in dorsolateral striatal medium spiny neurons (MSN) in mutant mice carrying full Shank3 deletion in exons 4–22 (Δe4–22^−/−^), which also exhibit decreased striatal spine density and altered striatal synapse postsynaptic density (Peça et al., 2011; Sala et al., 2015; Peixoto et al., 2016; Wang X. et al., 2016). Finally, BTBR T + Itpr3tf/J mice show impairments in mesolimbic and striatal synaptic dopamine D2 receptor signaling resulting in reduced dopamine neurotransmission. Reductions in pre-and post-synaptic adenosine A2A receptor function also indicate associations with altered dopamine neurotransmission (Squillace et al., 2014).

Overall, dopaminergic circuitry in the basal ganglia mediates rigid and sequential behavioral phenotypes associated with ASD. As dopamine-containing neurons and pathways are crucial in movement and sequencing behaviors, the regulation of the dopaminergic system may provide a valuable tool for modulating repetitive behaviors. Hence, basal ganglia circuits play an instrumental role in the regulation of compulsive and repetitive behavioral phenotype associated with ASD.

## Glutamatergic Signaling at Cortico-Striatal Synapses

Striatal glutamatergic synapses express synapse-associated protein 90/postsynaptic density protein 95 (SAP90/PSD95) associated proteins (SAPAP), which form scaffolding protein complexes involved in the regulation of neurotransmitters trafficking and targeting to the post-synaptic membrane (Wu et al., 2012). Mutations in synapse-associated protein 90/postsynaptic density protein 95-associated protein 3 (SAPAP3) that also binds to SHANK3 postsynaptic scaffolding protein is associated with stereotypic behaviors in mice (Sapap3^−/−^), such as compulsive self-grooming to the point of inducing lesions, which is rescued by Sapap3 re-expression in the striatum and optogenetic stimulation of lateral orbitofrontal cortex (Welch et al., 2007; Bienvenu et al., 2009; Burguière et al., 2013).

Sapap3 mutant mice exhibit glutamatergic transmission defects at cortico-striatal synapses and elevated mGluR5 signaling, leading to abnormal striatal output and stereotyped behavior, which is alleviated by mGluR5 inhibition (Ade et al., 2016). This suppression of mGluR5 possibly inhibits the direct basal ganglia pathway resulting in reduced repetitive behaviors (Conn et al., 2005). NMDA and AMPAR-dependent cortico-striatal synaptic transmission is also altered. Intriguingly, systemic administration of fluoxetine, a serotonin uptake inhibitor attenuates obsessive grooming in mutant mice (Welch et al., 2007).

## Endocannabinoid Signaling in Striatal Synapses

**Figure 4:**
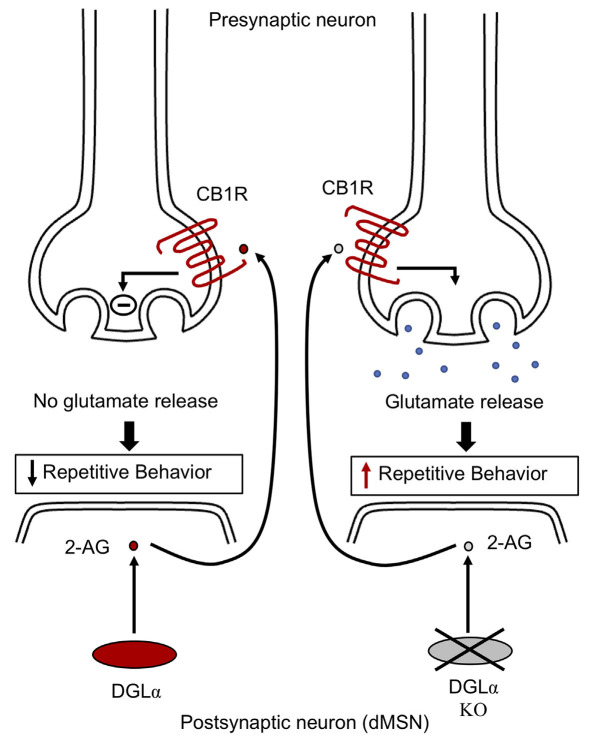
Endocannabinoid signaling in striatal neurons. DGLα synthesizes 2-AG in the postsynaptic neuron. Postsynaptic 2-AG activates presynaptic cannabinoid-1 receptor (CB1R). The activated CB1 receptor *via* feedback inhibition leads to suppression of glutamate release at MSN synapses, thereby relieving repetitive behavior. However, mice with knockout of DGLα exhibit decreased striatal 2-AG levels, resulting in unrestricted synaptic glutamate release *via* an absence of feedback inhibition, thereby leading to elevated grooming behavior in mice. Impaired endocannabinoid signaling is involved in the alteration of striatal activity, contributing to the development of repetitive behavior. CB1R, cannabinoid type 1 receptor; DGLα, diacylglycerol lipase alpha; 2-AG, 2-arachidonoyl glycerol; dMSN, direct pathway medium spiny neurons.

Endocannabinoid signaling plays a crucial part in modulating striatal synaptic transmission and in regulating stereotypic behaviors (Chen et al., 2011; Gremel et al., 2016). The abundant endocannabinoid, 2-arachidonoyl glycerol (2-AG), activates cannabinoid-1 receptor (CB1R), mediating suppression of glutamatergic release *via* feedback inhibition at direct and indirect medium spiny neuron (MSN) synapses (Kano et al., 2009). Synthesis of 2-AG in the postsynaptic neuron is mediated by diacylglycerol lipase alpha (DGLα) (Gao et al., 2010; Tanimura A. et al., 2010; Shonesy et al., 2014). Mice with DGLα knockout in direct-pathway MSN exhibit reduced levels of 2-AG in the striatum and absence of feedback inhibition mediated by 2-AG at glutamatergic direct-pathway MSN synapses, resulting in excessive glutamatergic drive in direct-pathway MSNs (Figure 3). In addition, DGLα deletion in direct-pathway MSNs does not change GABAergic synaptic transmission, suggesting that alterations to excitation/inhibition balance may contribute to increased direct-pathway MSN output, resulting in excessive grooming behavior (Figure 4). Furthermore, mice with regional DGLα deletions in the ventral striatum (nucleus accumbens) exhibit repetitive grooming behavior (Shonesy et al., 2018). Thus, 2-AG signaling impairment in direct pathway MSNs leads to circuit alterations and ASD behavioral phenotypes, such as repetitive self-grooming behavior (Figure 2).

Group1 mGluRs play a role in mobilizing endocannabinoids in the hippocampus, contributing to increased excitability. In FMR1 null mice, mGluR5 dependent LTD is absent at excitatory synapses of PFC and ventral striatum, which is moderated by endocannabinoid 2-arachidonoylglycerol (2-AG). The Homer scaffolding complex linking mGluR5 to DGLα is disrupted resulting in impairment of endocannabinoid mediated LTD at excitatory synapses. Application of CB1R antagonist rimonabant improves cognitive deficits in Fmr1 KO mice (Busquets-Garcia et al., 2013). Hence, endocannabinoid signaling contributes to increased excitability in FXS (Jung et al., 2012; Tang and Alger, 2015). Intriguingly, CB1, and CB2 receptor expression is upregulated in the brain of MeCP2 mutant mice. Treatment with cannabinoid cannabidivarin (CBDV) ameliorates memory deficits in MeCP2 mutant mice. CBDV also regulates BDNF, CB1, CB2 receptor levels, and PI3K/AKT/mTOR pathway which is dysregulated in MeCP2 deficient mice (Zamberletti et al., 2019). Hence, altered endocannabinoid signaling is associated with behavioral abnormalities in neurodevelopmental disorders.

## Astrocytic Calcium Signaling Regulating Striatal Circuitry

Astrocytes perform numerous functions, including maintenance of the blood-brain barrier, extracellular ion homeostasis, synapse formation, and regulation of synaptic transmission (Khakh and Sofroniew, 2015). Astrocytes also propagate intercellular Ca^2+^ waves upon stimulation and modulate neuronal function through Ca^2+^ dependent signaling (Bazargani and Attwell, 2016). Astrocytic Ca^2+^ signaling stimulates the release of gliotransmitters such as glutamate, GABA, ATP, and D-serine that regulate neuronal activity (Bazargani and Attwell, 2016). Astrocytes regulate extracellular levels of glutamate *via* transporters like GLT1, hence influencing excitatory and inhibitory neuronal balance (Wu et al., 2012). High levels of glutamate in the extracellular space lead to over-activation of glutamate receptors, i.e., neuronal excitotoxicity. Astrocytes protect against neurotoxicity by mediating glutamate clearance from synaptic space *via* glutamate uptake transporters, thereby modulating neuronal activity. Astrocytes also supply ATP that is crucial for the process of glutamate uptake. In astrocytes, glutamate is converted to glutamine which acts as a precursor for the resynthesis of neurotransmitters like glutamate/GABA in neurons. Further, glutamate in the synapse induces astrocytic Ca^2+^ increase that results in release of glutamate from astrocytes to adjoining neurons, stimulating NMDA receptors and iGluRs (ionotropic glutamate receptors), modulating their activity. Therefore, astrocytes have dual roles in maintaining glutamate release and uptake (Bazargani and Attwell, 2016; Mahmoud et al., 2019). Astrocytes also modulate synaptic GABA levels *via* GABA transporters (GAT) that mediates GABA uptake. Expression of synaptic GAT1 regulates GABA levels in the synapses, thereby modulating neuronal excitability. The rise in astroglial Ca^2+^ signaling leads to inhibition of neuronal activity. This is associated with elevated GABA levels in the synapse caused by decreases in astroglial membrane GAT levels *via* endocytosis into astrocytes. The membrane trafficking of GAT is regulated by Rab11, Rab family small GTPases. Rab11 suppression counteracts the decrease in neuronal activity by elevated astroglial Ca^2+^ levels *via* repressing GAT endocytosis. Therefore, astrocytes regulate activity of neuronal circuits (Zhang et al., 2017). Alterations in astroglial uptake processes or gliotransmitters release is implicated in the pathogenesis of neurological disorders including epilepsy and may contribute to the development of behavioral impairments in these disorders (Mahmoud et al., 2019).

**Figure 5:**
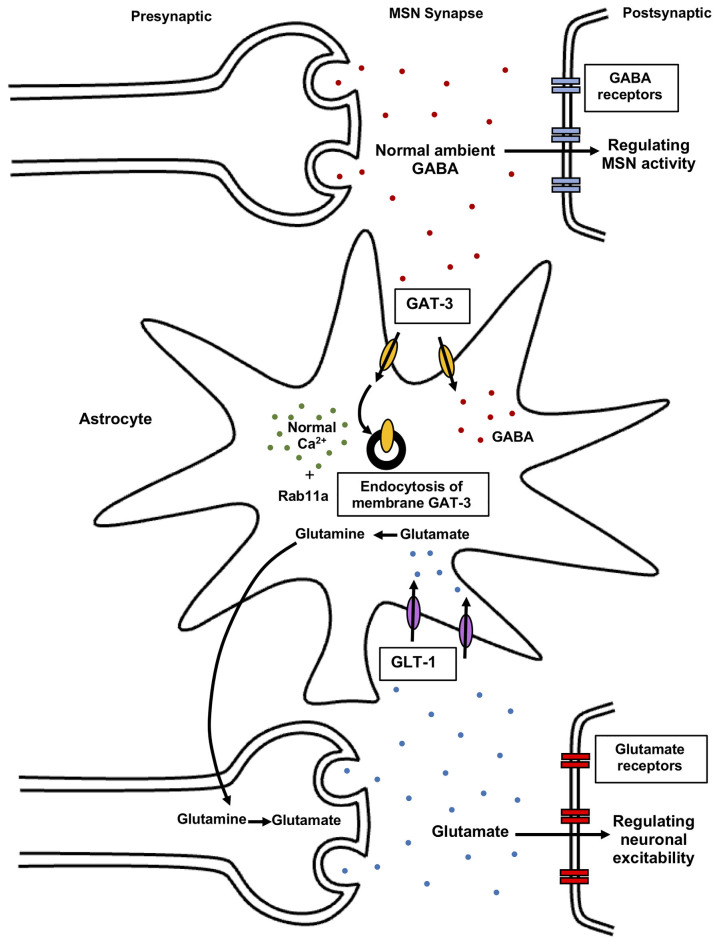
Astrocytic regulation of synaptic glutamate and GABA levels. Normal astrocytic Ca^2+^ signals modulate GAT-3 levels in the presence of Rab11a GTPase mediating GAT-3 endocytosis. As a result, controlled ambient GABA levels in the synapses regulate striatal MSNs activity, resulting in normal behavior. Reduced striatal astrocyte Ca^2+^ signaling contributes to elevated self-grooming behavior *via* altered striatal MSN activity. Astrocytes also regulate synaptic glutamate levels *via* transporters like GLT-1. Elevated glutamate levels in the extracellular space induce over-activation of glutamate receptors resulting in excitotoxicity. Astrocytes protect against this excitotoxicity by clearance of synaptic glutamate *via* glutamate uptake transporters. In astrocytes, glutamate is converted to glutamine which acts as a precursor for re-synthesis of glutamate in neurons, mediating both uptake and release of glutamate. Astrocytes regulate glutamate and GABA in the synapse, thereby modulating neuronal activity and behavior. GABA, gamma-aminobutyric acid; GAT-3, GABA transporter 3; GLT-1, glutamate transporter 1; Rab, small Rab GTPase.

Also, astrocytic dysfunction is implicated in stereotypic behaviors associated with neuropsychiatric disorders (Molofsky et al., 2012; Aida et al., 2015; Yu et al., 2018). Mutant mice with GLT-1 inducible deletion in astrocytes (GLAST^CreERT2/+^/GLT1^flox/flox^, iKO) display excessive self-grooming repetitive behavior resulting in self-induced injury. The knockout of astroglial GLT1 leads to alteration in the cortico-striatal synapse, suggesting glial dysfunction involvement in the pathophysiology of repetitive behaviors (Aida et al., 2015). In wild-type C57BL/6NTac mice, decreased astrocyte Ca^2+^ signaling in the striatum leads to increased stereotypic grooming behavior (Figure 2). In these experiments, wild-type C57BL/6NTac mice were injected with hPMCA2w/b construct to impair striatal astrocytic Ca^2+^ signals. The hPMCA2w/b construct consists of a w/b splice variant in human plasma membrane Ca^2+^-ATPases pump (hPMCA2) deficient in the cytosolic interaction domains (Yu et al., 2018). Membrane targeting of PMCA2 is determined by alternative splicing of protein cytosolic loop, in which “w” form (w splice variant) containing 45 amino acid residue insertion, display membrane localization of PMCA2. The b splice variant is generated at the COOH terminal site of the protein, an important regulatory region of the pump and its terminal sequence interacts with PDZ proteins (Chicka and Strehler, 2003). Astrocytes express the plasma membrane Ca^2+^ pump (PMCA2) that function to expel cytosolic Ca^2+^. The generated hPMCA2w/b mice exhibit excessive repetitive self-grooming behavior. Reduced astrocyte Ca^2+^ signaling decreases ambient GABA levels *via* enhanced GABA transporter 3 (GAT-3) activity (Figure 5). Also, Rab11a gene downregulation leads to increased GAT-3 functional activity, thereby reducing inhibition of MSNs in the striatum. The elevated self-grooming behavior is also observed in a mouse model of Huntington’s disease, R6/2 that is associated with decreases in astrocytic Ca^2+^ signals and alleviated by blocking astrocytic GAT-3. Hence, attenuated astrocytic Ca^2+^ signaling decreases striatal MSN inhibition, *via* altered GABA levels resulting in repetitive behavior (Yu et al., 2018) (Figure 5). Moreover, astrocytic GLT1 deficient mice show increased grooming, rearing, and jumping behavior, suggesting reduced synaptic glutamate clearance resulting in glutamatergic dysfunction underlying these behaviors (Jia et al., 2021). Hence, astrocytes regulate striatal activity and associated stereotypic behavior.

Further, mice with inactivation of *Tsc1* gene in astrocytes (*Tsc1*^GFAP^CKO) displays epilepsy, learning deficits, reduced GLT-1 protein expression, elevated levels of glutamate in the hippocampus, and impairment of hippocampus-LTP suggesting altered glutamate homeostasis and synaptic plasticity in a mouse model of Tuberous Sclerosis (Wong et al., 2003; Zeng et al., 2007).

Glial ephrin-A3 also plays an important role in modulating hippocampal activity. In the adult hippocampus, dendritic spines of pyramidal neurons express EphA4 tyrosine kinase receptor, the activation of which is dependent on ligand ephrin-A3, present in the perisynaptic processes of astrocytes, is involved in the regulation of dendritic spine morphology and synapse formation (Murai et al., 2003; Klein, 2009). Mice with a knockout of ephrin-A3 or EphA4 exhibits spine irregularities and results in increased expression of astroglial glutamate transporters GLT-1 and GLAST in the hippocampus. Hence, bidirectional signals between neuronal EphA4 and astroglial ephrin-A3 regulate spine morphology, glutamate transport, and excitatory synaptic function (Carmona et al., 2009; Filosa et al., 2009).

Neural circuit refinement is associated with experience-dependent synaptic pruning. In the cortex of ephrin-A2 knockout mice, experience-dependent removal of postsynaptic dendritic spines was mediated by activation of NMDA glutamate receptors, thereby leading to changes in adult neural circuits. Ephrin-A2 null mice also showed reduced glutamate transporters, contributing to increasing synaptic glutamate and promoting spine elimination (Yu et al., 2013).

Hence, astroglial expressed ephrin-A3 and ephrin-A2 in the hippocampus and cortex, respectively, have opposite effects on the modulation of glutamate transporters and spine morphology. Treatment interventions targeting astroglial ephrin-A3/A2 signaling may alter the expression of glutamate transporters and protect against glutamate excitotoxicity, maintaining the synapse structure and dynamics.

## Amygdala and Limbic Circuitry in Repetitive Behaviors

The amygdala is involved in the regulation of emotions, anxiety, and fear, as well as regulating repetitive behaviors. High levels of anxiety in rodents are accompanied by increased self-grooming behaviors, rescued by anxiolytic treatments (Kalueff and Tuohimaa, 2004a; Ahmari and Dougherty, 2015). Anxiety-related behavior in rats is correlated with reduced dopamine release in the amygdala and increased grooming episodes. In the medial nucleus of the amygdala (MeA), activation of vesicular glutamate transporter 2 (vGLUT2) expressing glutamatergic neurons increases repetitive self-grooming behavior (Figure 2), whereas activation of vesicular GABA transporter (VGAT)-positive GABAergic neurons represses self-grooming behavior in mice (Figure 3) (Hong et al., 2014). Also, injections of Orexin-B, a neuropeptide that regulates food intake, mood, and wakefulness in the central nucleus of the amygdala (CeA), lead to enhanced grooming frequency in hamsters. Orexin-B-induced grooming behavior is potentiated by infusion of NMDA receptor agonists (Alò et al., 2015). In the lateral amygdala, the Fmr1 KO mouse model shows synaptic defects including impaired mGluR-dependent LTP, and reduced AMPAR subunit, GluR1 surface expression (Suvrathan et al., 2010).

The basolateral nucleus of the amygdala (BLA) sends projections to the hippocampus and the prefrontal cortex (PFC; Obeso and Lanciego, 2011). Activation of glutamatergic projections from the basolateral amygdala (BLA) to the ventral hippocampus heightens self-grooming in mice (Felix-Ortiz and Tye, 2014) (Figure 2), while its inhibition leads to reduced locomotor activity, suggesting a crucial role for the ventral hippocampus in repetitive behaviors (Figure 3) (Bast et al., 2001; Zhang et al., 2002). Shank3 deficient rats show attention deficit and decreased synaptic plasticity in the hippocampal-medial prefrontal cortex pathway. Mouse models of Shank3 deletion also exhibit impaired synaptic plasticity in the hippocampus, associated with deficits in actin cytoskeleton remodeling, along with changes in NMDA glutamatergic receptors and mGluR-Homer scaffolding complex, resulting in abnormalities in cortico-striatal circuits underlying repetitive behaviors (Bozdagi et al., 2010; Duffney et al., 2013; Kouser et al., 2013; Wang X. et al., 2016). In addition, the Shank postsynaptic protein scaffold helps regulate synaptic transmission at hippocampal Schaffer Collateral-CA1 synapses (Shi et al., 2017). Further, altered synaptic transmission at thalamo-amygdala circuits is associated with obsessive self-grooming behavior in rodents (Ullrich et al., 2018).

The hypothalamus is another limbic brain region involved in regulating numerous behaviors, including self-grooming in rodents (Qualls-Creekmore and Münzberg, 2018). The hypothalamic paraventricular nucleus and the dorsal hypothalamus are associated with grooming behavior observed by local electrical stimulation in the hypothalamus that induces self-grooming in rats. The paraventricular nucleus projects to the posterior dorsal part of the medial amygdala (MeApd) which is involved in self-grooming behavior (Roeling et al., 1993). Lateral hypothalamic glutamatergic neurons adjacent to the MeApd play roles in repetitive self-grooming behaviors in mice (Figure 3). Moreover, MeApd also projects to the medial hypothalamus (Hong et al., 2014). Finally, the central nucleus of the amygdala (CeA) and MeA projects to the BNST that connects the amygdala and hypothalamus (Heimer et al., 2007). Hence, the limbic system, incorporating the amygdala, hippocampus, hypothalamus, and basal ganglia regions, play important roles in regulating repetitive behaviors.

## Neuroanatomy of ASD

Magnetic resonance imaging (MRI) studies in humans have contributed to the understanding of the neuroanatomical basis of ASD, such as a period of early brain overgrowth in autism, particularly in frontal, temporal and cingulate cortices, hippocampus, cerebellum, and amygdala (Palmen and van Engeland, 2004; Bauman and Kemper, 2005; Courchesne et al., 2007; Amaral et al., 2008). Further, atypical functional connectivity between caudate and cortical areas has been observed in autistic subjects (Turner et al., 2006). These findings match neuroanatomical alterations observed in several of the mice models discussed above, which also show alterations to the hippocampal commissure, decreased frontal-cortical, occipital, and thalamic gray matter volume along with reduced cortical thickness (Wahlsten et al., 2003).

Neuroimaging studies also suggest an association of repetitive behaviors, with the volume of basal ganglia areas, such as the caudate-putamen (Sears et al., 1999; Calderoni et al., 2014). Autistic individuals show significantly larger right caudate and putamen volumes compared to matched controls. Moreover, total putamen and right caudate volumes reveal a positive association with ADI-C domain repetitive behavior scores (Hollander et al., 2005). Neuroimaging of individuals with fragile X syndrome (FXS) also exhibit altered gray matter volume in the caudate and white matter of the ventral frontostriatal pathway (Haas et al., 2009; Hallahan et al., 2011). Moreover, imaging studies of RTT individuals show reduced caudate nucleus and midbrain volumes (Casanova et al., 1991; Reiss et al., 1993; Subramaniam et al., 1997).

The medial frontal gyri, right fusiform gyrus, and left hippocampal volumes are also enlarged in autistic groups (Rojas et al., 2006; Verhoeven et al., 2010). The increased regional brain volumes show a positive correlation with stereotypic behaviors; however, the decreased volume of the cerebellum in autistic subjects shows a negative correlation with repetitive behavioral measures (Rojas et al., 2006). One study on autistic children demonstrated a positive association of repetitive behavior and frontal lobe volume and a negative association with cerebellar vermis volume (Pierce and Courchesne, 2001). Also, developmental studies in rodents and non-human primates show that damage to the amygdala, hippocampus, and temporal cortex induces ASD-like behaviors such as stereotypies (Bachevalier and Loveland, 2006). Early in life, amygdala and hippocampal lesions result in self-directed and stereotypic head twisting behaviors in juvenile monkeys (Bauman et al., 2008).

The anterior cingulate cortex (ACC) is also implicated in repetitive behaviors in ASD (Thakkar et al., 2008). An fMRI study in high-functioning autistic individuals revealed a negative correlation of repetitive/restricted behaviors with ACC and posterior parietal activation implicating frontal-striatal circuitry in stereotyped behaviors (Shafritz et al., 2008). Additional consistent neuroimaging findings are required to understand the neural circuitry of stereotypic behaviors in neurodevelopmental disorders.

Imaging studies in preclinical animal models are limited and research in this area is still ongoing (Wilkes and Lewis, 2018). There are a few MRI studies that have utilized diffusion tensor imaging (DTI) and functional MRI (fMRI) in animal models of repetitive behaviors (Ellegood et al., 2010, 2013; Dodero et al., 2013; Squillace et al., 2014; Haberl et al., 2015; Allemang-Grand et al., 2017). Mice with hemizygous (−/Y), heterozygous (−/+) and homozygous (−/−) *Mecp2* mutation show enlarged cerebellar volume, including the vermis, cerebellar cortex region, and smaller cortical volumes including somatosensory, frontal, motor, and cingulate regions. Also, *Mecp2* hemizygous male mice (−/Y) exhibit increased brainstem volume and reduced volumes in the striatum, thalamus, frontal cortex, and corpus callosum. These studies correlate with imaging findings in individuals with Rett syndrome (Dunn et al., 2002; Carter et al., 2008; Ellegood et al., 2015; Allemang-Grand et al., 2017).

MRI imaging in *Fmr1* KO mice reveals decreased cerebellar nuclei and striatal volumes (Ellegood et al., 2010). Also, diffusion tensor MRI and functional MRI (fMRI) studies show changes in structural connectivity of the corpus callosum and functional connectivity between cortical regions such as visual, somatosensory, auditory, and motor regions (Haberl et al., 2015). MRI analysis of 16p11.2 CNV mice demonstrates volumetric alterations in brain regions including basal forebrain, hypothalamus, midbrain, and superior colliculus (Horev et al., 2011). Additionally, 16p11^+/–^ pups show reduced brain volume at postnatal day 7, while the relative volume i.e., normalized to total brain volume of nucleus accumbens (NAc) and globus pallidus (GP) regions is increased. Structural abnormalities in cortical areas are also observed in 16p11^+/–^ pups (Portmann et al., 2014). Adult heterozygous 16p11.2 mice after controlling for total brain volume show neuroanatomical alterations in different brain regions including increased midbrain, hypothalamus, superior colliculus volumes, and reduced striatal volume (Ellegood et al., 2015). Mice with chromosome 15 mutations, particularly with duplication of the 15q11–13 region show reduced relative volumes for different brain areas like basal forebrain, midbrain, hypothalamus, and thalamus (Ellegood et al., 2015).

Decreases in parvalbumin-containing interneurons in the medial prefrontal cortex are observed in ASD individuals (Hashemi et al., 2017). *Parvalbumin* knockout mice show ASD behavioral phenotypes, such as deficits in social interaction behaviors, ultrasonic vocalizations, and higher-order reversal learning in the T-maze assay (Wöhr et al., 2015). An MRI study of juvenile *Parvalbumin* knockout mice revealed reduced cortical volume and increased cerebellar volume. However, these anatomical alterations are not consistent in adult *Parvalbumin* knockout mice (Wöhr et al., 2015). Additional studies are required for elucidating other repetitive behaviors and brain region structural alterations in this mouse model. *In utero* VPA exposed rats exhibit decreased total brain volume, relative cortical and brainstem volumes, and hippocampus volume (Frisch et al., 2009; Petrenko et al., 2013).

BTBR mice exhibit reduced cerebral white and gray matter, ventricular volumes, and larger olfactory, brainstem, and cerebellum volumes compared to C67BL/6 mice (Ellegood et al., 2013). An fMRI study of BTBR mice showed decreased bilateral functional connectivity for cingulate, striatum, insular, motor cortex, and reduced striatal-thalamic connectivity. However, hippocampus, temporal and occipital areas show increased interhemispheric connectivity in BTBR mice (Sforazzini et al., 2016).

Molecularly, scaffolding proteins, glutamate receptor-interacting proteins 1/2 (Grip1/2), plays a role in AMPAR trafficking and its absence contributes to cerebellar LTD deficit in cultured Purkinje cells and social preference changes in cell-specific Grip1/2 mutant mice (Takamiya et al., 2008; Mejias et al., 2011). Grip1/2 KO mice exhibit repetitive grooming with no changes in social interaction and anxiety, normal mEPSCs but weakened mGluR-LTD at the parallel fiber-PC synapses and altered expression of arc, mGluR5, phosphorylated P38 and AKT in the Purkinje cells. So, defects in Grip1/2 mediating AMPAR trafficking at cerebellar Purkinje cells along with impaired mGluR5 signaling in cerebellum results in the pathogenesis of repetitive behaviors (Mejias et al., 2019). Mice with conditional *Pten* inactivation in Purkinje cells show stereotyped jumping and decreased motor learning with a structural aberration in PC dendrites, axons, reduced excitability, altered parallel fiber and climbing fiber synapses (Cupolillo et al., 2016). Further, the mouse model of Tuberous Sclerosis with *Tsc2* loss in Purkinje cells (Tsc2f/−; Cre mice) displays increased marble burying repetitive behavior and Purkinje cell dysfunction, suggesting Purkinje cell loss contribution to ASD phenotype (Reith et al., 2013). Therefore, the cerebellum, particularly Purkinje cells and associated signaling pathways play important role in the regulation of repetitive behaviors.

Post-mortem studies of autistic cases have also implicated many of these same brain regions. Purkinje cells (PC) in the cerebellum are consistently altered in neuropathological analyses of ASD brain samples (Fatemi et al., 2002; Palmen and van Engeland, 2004; Whitney et al., 2008). However, the limitation of imaging studies includes poor tissue quality and small sample sizes, as well as an analysis of samples from adult brains which does not provide information regarding development (Amaral et al., 2008).

Overall, neuroanatomical alterations are largely found in frontal, temporal cortical regions, basal ganglia areas, and cerebellum in human studies and mouse models showing repetitive behaviors (Ellegood et al., 2010, 2013, 2015; Portmann et al., 2014; Haberl et al., 2015; Wöhr et al., 2015). Basal ganglia areas such as striatum and globus pallidus show volumetric alterations related to stereotyped behaviors (Ellegood et al., 2010, 2013, 2015; Portmann et al., 2014). Associations between repetitive behavioral phenotypes and changes in specific brain region structural and functional aspects require additional studies in animal models of ASD and other neurodevelopmental disorders.

## Anxiety and Repetitive Behaviors

ASD is associated with anxiety disorders and the prevalence estimates of anxiety in ASD individuals vary widely from 22% to 84% (van Steensel et al., 2011; Lai et al., 2014; Vasa and Mazurek, 2015; Lever and Geurts, 2016; Russell et al., 2016; Nimmo-Smith et al., 2020). There is also a significant relationship between anxiety and restricted/repetitive behaviors in the ASD population (Gotham et al., 2013; Stratis and Lecavalier, 2013; Postorino et al., 2017; Russell et al., 2019; Baribeau et al., 2020). Association of anxiety with ritualistic behaviors is related to abnormal sensory gating suggesting altered sensory processing (Green et al., 2012; Mazurek et al., 2013; Lidstone et al., 2014).

Grooming behavior reflects repetitive, stress-coping behavior and complex interplay with anxiety and motor activity in rodents (Kalueff and Tuohimaa, 2005a; Lewis et al., 2007; O’Leary et al., 2013). Some ASD mouse models demonstrate both anxiety and repetitive behaviors. In a mouse model of Rett syndrome, deletion of MeCP2 in the basolateral amygdala causes increases anxiety and learning deficits (Adachi et al., 2009). The increased grooming behavior in EphrinA2/A3 double KO mice may correlate with sensorimotor gating deficits and abnormal sensory processing as a result of exposure to novel environments (Wurzman et al., 2015). The Shank1 mice model of ASD manifests mild anxiety and repetitive behavior (Hung et al., 2008). ASD mice models with FMR1, PTEN, UBE3A, and GABRB3 mutations exhibit learning deficits, stereotypic behaviors, and anxiety phenotypes (Jiang et al., 2010; Tanaka et al., 2012; Gandhi et al., 2014; Clipperton-Allen and Page, 2015; Zieba et al., 2019). Additionally, the BTBR mouse model of autism displays anxiety traits and repetitive behaviors (McFarlane et al., 2008; Pobbe et al., 2011). In contrast, some mouse models exhibiting repetitive behaviors do not show anxiety-like behaviors or are not reported in some cases. Mouse models including mutations in CNTNAP2, neuroligin1, the oxytocin receptor, and 16p11.2 chromosomal deletions do not display anxiety behaviors or are not reported in some studies (Peñagarikano et al., 2011; Crawley, 2012; Kazdoba et al., 2016). Thus, future studies are required to elucidate the anxiety phenotype along with the repetitive behavior in different rodent models of ASD.

Acute and chronic stress plays a role in alterations of grooming activity (Katz and Roth, 1979; Fentress, 1988; Kalueff and Tuohimaa, 2004b; Komorowska and Pellis, 2004). For instance, C57BL/6J male mice following chronic social defeat stressors, display disorganized cephalo-caudal grooming patterning and induces anxiety (Veenema et al., 2003; Kinsey et al., 2007; Denmark et al., 2010). Additionally, Wistar rats exposed to the lightbox show increased grooming frequency and duration as compared to rats exposed to the dark box. The light-dark paradigm helps in assessing stress levels in rats *via* counting the number of defecation boli and urination spots, indicating more anxiety in rats exposed to the lightbox. This may suggest that stress and anxiety may affect grooming activity and its microstructure in rodents (Kalueff and Tuohimaa, 2005b, 2004b). Surprisingly, some inbred mouse strains demonstrate high or low grooming in response to anxiety. The BALB/c mice show increased grooming compared to 129S1 mice. The high grooming in BALB/c mice may correlate with increased anxiety as assessed by high defecation boli scores, one of the stress markers in rodents. In contrast, 129S1 mice show low-grooming and high anxiety levels, indicating that different rodent strains exhibit variation in anxiety-induced behaviors (Kalueff and Tuohimaa, 2004a, 2005a). Anxiolytics like bupropion (noradrenaline and dopamine reuptake inhibitor), fluvoxamine (SSRI), diazepam (benzodiazepine), and imipramine (tricyclic antidepressant) decreased marble burying and digging behavior in mice (Hayashi et al., 2010). Further, minocycline ameliorates marble-burying behavior and correlates with proper dendritic spines maturation in Fmr1 KO mice (Dansie et al., 2013). Studies on marble-burying are controversial as some indicate that marble-burying correlates with anxiety whereas others indicate that it reflects repetitive digging (Njung’e and Handley, 1991; Thomas et al., 2009; Taylor et al., 2017; de Brouwer et al., 2019). Minocycline also alleviates aberrant grooming behavior and modulates hippocampal GABA levels in rats (Zhang et al., 2019).

Neuropsychiatric and neurodevelopmental disorders including autism, OCD, schizophrenia, and anxiety share some symptoms and overlap in common pathological genes, circuits, and mechanisms (Shavitt et al., 2006; Kalueff and Nutt, 2007; Kalueff et al., 2008; Szechtman et al., 2017). For instance, GABAergic activity alterations are associated with anxiety, depression, and autistic phenotypes, indicating common underlying neural pathology (Persico and Bourgeron, 2006; Kalueff and Nutt, 2007). Altered GABA receptor activity by anxiolytic (GABA enhancing) and anxiogenic (GABA inhibiting) drugs correlates with a decrease and increase in stress-induced grooming behavior. This may indicate that these drugs regulate the strength of the anxiogenic stimuli perception and grooming behavior (Kalueff and Tuohimaa, 2005c; Nin et al., 2012; Xu et al., 2012; Kalueff et al., 2016). Similarly, BDNF and serotonin transporter (SERT) gene has been linked to cognitive deficits, anxiety, depression, schizophrenia, OCD, and autism (Devlin et al., 2005; Hu et al., 2006; Kaufman et al., 2006; Kalueff et al., 2007; Kas et al., 2007; Moy and Nadler, 2008). Rodents manifest heightened grooming behavior in response to changes in the environment by stressful and/or anxiogenic stimuli (Gispen and Isaacson, 1981; Florijn et al., 1993; Gargiulo and Donoso, 1996). Dopaminergic activity in the basal ganglia pathways likely mediates the stress-coping grooming behavior (Spruijt et al., 1986, 1992; Cools et al., 1988; Kametani, 1988; Reis-Silva et al., 2019). Anxiety-like behaviors correlate with decreased dopamine release in PFC, substantia nigra, and amygdala of rats spending more time self-grooming induced by stress on exposure to the elevated plus-maze (EPM). This suggests that self-grooming is associated with reward systems and may be reflective of de-arousal activity instead of a direct response to anxiety (Homberg et al., 2002). Additionally, serotonin plays a role in regulating stress-coping behavior such as self-grooming (Houwing et al., 2019). Hence, rodent grooming may represent one method for stress reduction or de-arousal, instead of directly involved in the stress response (Estanislau et al., 2013, 2019).

Also, several common brain regions have been associated with anxiety and repetitive behavioral disorders, particularly the amygdala and PFC. For instance, muscimol (GABA agonist) infusion into the basolateral nucleus of the amygdala and PFC decreases anxiety in rats (Shah et al., 2004; Bueno et al., 2005). Intriguingly, muscimol injection into BNST (extended amygdala), a region that regulates innate fear responses leads to decreased self-grooming behavior in rats (Xu et al., 2012). Additionally, GABAergic neurons in the MeApD region reduce self-grooming behavior (Hong et al., 2014). Further, injections of GABA-A receptor antagonist bicuculline into the basolateral amygdala increases anxiety in rats (Sajdyk and Shekhar, 2000). In the MeApD region, glutamatergic neurons promote stereotypic self-grooming (Hong et al., 2014). Alterations in GABA, serotonin, kainate, and glutamate receptor densities in various amygdala nuclei correlate with anxiety-like behavior in some inbred mouse strains (Yilmazer-Hanke et al., 2003; Caldji et al., 2004). Amygdala stimulation leads to increases in anxiety and facilitates compulsive behaviors (McGrath et al., 1999). In the case of OCD, basolateral amygdala projections to medial PFC modulate repetitive checking behavior in rodents (Sun et al., 2019). One of the brain regions involved in stress coping responses, the periaqueductal gray (PAG) and its pathways, influences self-grooming behavior (Bandler et al., 2000). Alteration in striatal neurons, CeA and mPFC projections to the PAG region may affect self-grooming behavior (Spruijt et al., 1992; Floyd et al., 2000). Increased expression of c-fos is observed in the hippocampus, hypothalamus, PFC after administration of anxiogenic drugs, and hypothalamic injection of GABAergic anxiolytic drugs reduces anxiety in rats (Jardim and Guimarães, 2001; Singewald et al., 2003). Hence, regulated GABAergic activity and consequent excitatory neurotransmission in these brain regions are critical for the modulation of anxiety and repetitive behaviors, indicating overlapping circuits in anxiety and repetitive behaviors.

However, further studies are required to ascertain regional and circuit differences between anxiety-induced and repetitive self-grooming behavior. Investigations of animal models displaying both anxiety and repetitive behavior simultaneously or induction of one disorder by another will help in providing innovative insight into the common and specific neural alterations underlying these disorders.

## Summary

Animal models of neuropsychiatric and neurodevelopmental disorders such as autism have provided relevant knowledge on the neuronal circuitry and receptor targets implicated in the etiology and pathophysiology of repetitive behaviors. Several brain regions and neural circuits including cortico-basal ganglia-thalamic circuits, limbic circuits, prefrontal cortex, cerebellum, hypothalamus, and striatum are involved in the regulation of core autistic behaviors. Genetic mutations and environmental risk factors resulting in the presentation of repetitive behaviors in rodent models involve multiple cellular, molecular, and network factors. The majority of ASD alterations involve excitatory glutamatergic, inhibitory GABAergic, serotonergic and dopaminergic neurons, receptors, neurotransmitters, neuronal migration, and spine densities resulting in changes in signaling pathways and synaptic activity which may converge on common neural circuits (Golden et al., 2018).

Genome-wide association studies (GWAS) have indicated various ASD risk genes including neuronal cell adhesion molecules (neurexins, neuroligins, CNTNAP), postsynaptic scaffolding proteins (Shanks, SAPAP), neurotransmitter signaling and trafficking (Glutamate, GABA, EphA3), and molecules involved in protein synthesis in the brain (Fmr1, TSC, MeCP2) (Stearns et al., 2007; Tabuchi et al., 2007; Hung et al., 2008; Samaco et al., 2008; Etherton et al., 2009; Radyushkin et al., 2009; Peñagarikano et al., 2011; Peça et al., 2011; Silverman et al., 2011; Casey et al., 2012; Eadie et al., 2012; Schmeisser et al., 2012; Grayton et al., 2013; Monteiro and Feng, 2017; Wang et al., 2017; Zerbi et al., 2018). Many of the autism risk genes encode for proteins involved in excitatory glutamatergic signaling, converging at excitatory synapses (Peça et al., 2011; Qiu et al., 2012). For instance, Shank3 forms a scaffolding complex comprised of SAPAP that also interconnects with ephrins/Ephs and neurexin/neuroligin complexes (Qiu et al., 2012). This suggests that alterations in these molecules may converge on common synaptic and circuit mechanisms underlying autistic behavioral phenotypes. Understanding the mechanisms by which these factors affect neuronal circuits will provide insight into relevant targets of sensorimotor repetitive behaviors.

Although ASD etiological heterogeneity leads to complex and sometimes divergent behavioral outcomes in affected populations, a large literature exists, including neuroimaging studies, that have determined the crucial role of cortico-basal ganglia and limbic circuit alterations in mediating stereotypic behaviors. Altogether, common neural modifications in specific pathways and neural circuits lead to the emergence of repetitive behaviors in ASD. Inconsistencies in some studies and factors influencing generality of the repetitive behavioral findings may be related to sample, environment, and experimental heterogeneity. Future research integrating disparate findings hold immense potential to ascertain the involvement of common neural changes converging at the level of circuit alterations in neurodevelopmental disorders. More detailed work with additional animal models is required to dissect the molecular and neuroanatomical alterations in other pathways and brain regions implicated in repetitive behavioral phenotypes, to identify potential targets and treatment strategies for attenuating repetitive behaviors in affected individuals. Finally, early interventions for repetitive behaviors hold great promise for improving the quality of life for affected individuals.

## Future Directions and Limitations

The scope of this review is narrowed to neural mechanisms underlying lower-order repetitive behaviors in rodent models of ASD. Most of the literature in rodent models of ASD discusses lower-order stereotyped sensory-motor behaviors. However, some studies address higher-order insistence on sameness behaviors, such as circumscribed interests and resistance to change in a few rodent models. Future studies are required to evaluate common underlying molecular and circuit alterations in repetitive and restricted behaviors in autism. Further, characterization of both repetitive motor behaviors and insistence on sameness behaviors should be performed in different rodent models of ASD and other neurodevelopmental disorders to increase their translational value and to identify overlapping neurobiological alterations underlying these behaviors.

Although the studies reviewed here contribute to our understanding of the underlying neural alterations in rodent models displaying robust repetitive behaviors, the relation of such alterations with repetitive behavioral expression is unresolved. A focus of most investigations has been on the pathophysiology of mutations resulting in the expression of general ASD phenotype and rescuing the core ASD behavioral deficits rather than focusing exclusively on repetitive behaviors. Future findings targeting specific brain regions and focusing on neural alterations elemental to repetitive behaviors solely, while controlling for other behaviors, will provide a better understanding of how individual genetic and environmental changes converge at molecular and circuit levels to mediate repetitive behaviors. Alternatively, the generation of mutant rodent models with a targeted knockout of susceptibility genes in circumscribed brain regions may help in clarifying particular behavioral phenotypes. For instance, in NL3 mice, inhibition is elevated in the somatosensory cortex, whereas AMPAR mediated excitation is heightened in the CA1 hippocampal region (Etherton et al., 2011). Consequently, the specific neural circuitry associated with particular cognitive and behavioral components in ASD remains to be fully dissected. Regardless of these challenges, common circuits and molecular alterations provide a basis for understanding ASD etiological factors and behavioral abnormalities.

Also, very few studies have incorporated different methodological approaches to elucidate changes fundamental in mediating repetitive behaviors in rodents (Squillace et al., 2014; Wöhr et al., 2015; Sforazzini et al., 2016). A combination of different methodological approaches such as neuroimaging, histological and molecular analysis may provide a more comprehensive understanding of alterations in specific brain regions and their neural projections primarily mediating repetitive behaviors in rodent models of ASD. Also, future studies incorporating both male and female rodent models may help in elucidating any gender differences in brain structure and function associated with repetitive behaviors. Another important requirement is to evaluate molecular and circuit modifications fundamental to repetitive behaviors in other neurodevelopmental and neuropsychiatric disorders. Corroboration of findings across varied rodent models displaying repetitive behaviors may illuminate similar and dissimilar changes in brain pathways underlying these disorders.

A somewhat underexplored therapeutic avenue in rodent models is environmental enrichment (EE), which attenuates the repetitive behaviors in models of ASD. The EE reduces repetitive behaviors in deer mice by elevating indirect basal ganglia pathway function *via* increasing neuronal activation and dendritic spine densities in the subthalamic nucleus (STN) and globus pallidus (GP) (Bechard et al., 2016). However, mechanisms by which environmental enrichment alters repetitive behavior and correlations with structural, functional, and molecular modifications in brain regions demand a detailed investigation. Also, investigations of the effectiveness of environmental enrichment in attenuating repetitive behaviors should be extended to different rodent models of repetitive behavioral and neurodevelopmental disorders. This may help in probing the efficacy of environmental enrichment concerning repetitive behaviors.

Pharmacologically, systemic and local applications of glutamatergic inhibitors, GABAergic, serotonergic and dopaminergic agents have varied effects in different brain regions and circuits mediating repetitive behaviors. However, it remains to be determined whether these agents are applicable for alleviating behaviors beyond lower-order motor stereotypies in rodent models. Further research is required to ascertain if these various receptor agents also play a role in higher-order stereotypies in rodent models. Also, investigating the cross-over effects of these agents in different neural pathways may help to understand the underlying cellular and molecular pathologies concerning repetitive behaviors.

Also, future research studying overlapping or common pathways underlying stress, anxiety, and repetitive behaviors may provide some critical insight into targets directed towards these behavioral domains.

This review summarizes findings on molecular, signaling pathways, circuit, and neuroanatomical alterations in rodent models of ASD displaying robust repetitive behaviors. These findings emphasize important molecular, structural, and functional connectivity changes in brain regions like the prefrontal cortex, basal ganglia structures, limbic areas, and cerebellum, suggesting a major role of cortical-basal ganglia circuits. Besides, signaling pathways involving different neurotransmitters and their receptors such as glutamate, GABA, serotonin, and dopamine are also involved in the pathophysiology of stereotypic motor behaviors. Understanding the hierarchy of changes in different brain regions molecular, structure, function, and connectivity aspects mediating repetitive behaviors in rodent models will provide an important platform for translational study.

Last, comparative research involving human clinical population and animal models of ASD and other neurodevelopmental disorders holds enormous potential for unraveling the underlying neural alterations mediating repetitive behaviors and identifying directed pharmacological and circuit-based targets for treatment interventions.

## Author Contributions

TG and CL wrote the review article. All authors contributed to the article and approved the submitted version.

## Conflict of Interest

The authors declare that the research was conducted in the absence of any commercial or financial relationships that could be construed as a potential conflict of interest.
